# Rubisco small subunit (RbCS) is co-opted by potyvirids as the scaffold protein in assembling a complex for viral intercellular movement

**DOI:** 10.1371/journal.ppat.1012064

**Published:** 2024-03-04

**Authors:** Li Qin, Hongjun Liu, Peilan Liu, Lu Jiang, Xiaofei Cheng, Fangfang Li, Wentao Shen, Wenping Qiu, Zhaoji Dai, Hongguang Cui

**Affiliations:** 1 Key Laboratory of Green Prevention and Control of Tropical Plant Diseases and Pests (Ministry of Education) and School of Tropical Agriculture and Forestry, Hainan University, Haikou, China; 2 State Key Laboratory for Biology of Plant Diseases and Insect Pests, Institute of Plant Protection, Chinese Academy of Agricultural Sciences, Beijing, China; 3 College of Plant Protection/Key Laboratory of Germplasm Enhancement, Physiology and Ecology of Food Crops in Cold Region of Chinese Education Ministry, Northeast Agricultural University, Harbin, China; 4 Institute of Tropical Bioscience and Biotechnology, Chinese Academy of Tropical Agricultural Sciences, Haikou, China; 5 Center for Grapevine Biotechnology, William H. Darr College of Agriculture, Missouri State University, Mountain Grove, United States of America; University of Florida, UNITED STATES

## Abstract

Plant viruses must move through plasmodesmata (PD) to complete their life cycles. For viruses in the *Potyviridae* family (potyvirids), three viral factors (P3N-PIPO, CI, and CP) and few host proteins are known to participate in this event. Nevertheless, not all the proteins engaging in the cell-to-cell movement of potyvirids have been discovered. Here, we found that HCPro2 encoded by areca palm necrotic ring spot virus (ANRSV) assists viral intercellular movement, which could be functionally complemented by its counterpart HCPro from a potyvirus. Affinity purification and mass spectrometry identified several viral factors (including CI and CP) and host proteins that are physically associated with HCPro2. We demonstrated that HCPro2 interacts with both CI and CP *in planta* in forming PD-localized complexes during viral infection. Further, we screened HCPro2-associating host proteins, and identified a common host protein in *Nicotiana benthamiana*–Rubisco small subunit (NbRbCS) that mediates the interactions of HCPro2 with CI or CP, and CI with CP. Knockdown of *NbRbCS* impairs these interactions, and significantly attenuates the intercellular and systemic movement of ANRSV and three other potyvirids (turnip mosaic virus, pepper veinal mottle virus, and telosma mosaic virus). This study indicates that a nucleus-encoded chloroplast-targeted protein is hijacked by potyvirids as the scaffold protein to assemble a complex to facilitate viral movement across cells.

## Author summary

*Potyviridae* is the largest family of RNA viruses in the plant kingdom, consisting of genetically diverse members that adversely affect agriculturally and economically important crops. However, viral and host components in the local movement of potyvirids, an essential step for viruses to spread through the whole plant, are still not fully understood. Thus far, three viral factors (P3N-PIPO, CI and CP) and several host proteins are known to be engaged coordinately in this event. Here, we found that another viral protein, HCPro2, also aids a potyvirid in the intercellular movement. HCPro2 facilitates the virus to move through plasmodesmata (PD), the gate between two plant cells, by forming a complex with CI and CP. More intriguingly, we found that NbRbCS, one of the most abundant proteins in a plant cell, interacts with all the above viral factors in mediating viral movement across plant cells. Reduction of *NbRbCS* levels greatly impairs the intercellular movement of four tested potyvirids. These data suggest that a common chloroplast protein is co-opted as a pro-viral factor in assembling a complex for viral movement. This finding provides a new insight in our understanding of potyvirids’ movement in plants.

## Introduction

Plasmodesmata (PD) are plasma-membrane-lined nanochannels that cross rigid cell wall between adjacent cells, allowing the exchange of signals and resources among cells for developmental regulation and stress responses in higher plants [1–3]. Functional plasmodesmata are also found in bryophytes [4,5]. Plant viruses, as the obligate intracellular parasites, take full advantage of PD to spread intercellularly to establish systemic infection. However, the small aperture of PD allows small molecules to diffuse, but physically restricts the passage of macromolecules or macromolecular complexes such as viral ribonucleoprotein complexes (vRNPs) and virions [1,6,7]. To overcome the barrier, plant viruses encode diverse types of movement proteins (MPs) that interact with host proteins to modify PD to translocate vRNPs or virions [7–11]. Based on the characteristics of MPs and their interactions with PD, three modes of cell-to-cell movement are assigned to different plant viruses, and herein readers are directed to several excellent reviews [7,9,11–13]. However, the cell-to-cell movement for viruses in the *Potyviridae* family (potyvirids), representing the largest group of plant-infecting RNA viruses, has not been definitively categorized [7].

All potyvirids excluding bymoviruses possess one positive-sense, single-stranded RNA genome (~ 9.7 kb), which contains a long, full-genome open reading frame (ORF) and another relatively short ORF (PIPO) embedded in P3-coding region [14,15]. PIPO becomes translatable in frame with the coding region of P1 through the N-terminus of P3 (P3N) from viral genomic subpopulation, which is produced by viral RNA polymerase (NIb) slippage during viral replication [16,17]. Upon translation, two different polyproteins are proteolytically processed by virus-encoded proteases into 10 to 12 mature units [18,19]. None of them is annotated as MP, whereas three factors, P3N-PIPO (a translational fusion of P3N with PIPO), CI (cylindrical inclusion protein), and CP (coat protein), are known to regulate viral intercellular trafficking in a coordinated manner.

P3N-PIPO is a PD-localized viral factor, facilitating its own cell-to-cell movement [10,20,21]. Disrupting the generation of P3N-PIPO in different potyvirids restricts viral cell-to-cell movement but does not affect viral replication [22–25]. CI is a multifunctional viral protein [26]. Accumulating genetic evidence assigns an independent role for CI in viral intercellular movement [26–28]. CI is recruited to PD via an interaction with P3N-PIPO, and forms conical structures that anchor to and extend through PD [7,20,25]. The CI conical structures bind CPs or virions to aid viral intercellular passage [29–31]. Artificial mutation in CP that disrupts viral particle assembly compromises intercellular spread as well [32–35], suggesting that viral cell-to-cell movement occurs in the form of virion [7]. Helper component-protease (HCPro) is another multifunctional protein, and its function in RNA silencing suppression (RSS) was well-studied [36]. HCPro likely participates in cell-to-cell movement: i) HCPro of a virus in *Potyvirus* genus (potyvirus) has the capacity of trafficking between cells and increasing the size exclusion limit (SEL) of PD [37]; ii) HCPro stabilizes CP and enhances the yield of virions [38,39], suggesting its indirect role in viral intercellular movement [7]; iii) HCPro or CI of potato virus A (PVA) forms a protrusion at one end of virion [31,40]. Nevertheless, the connections between HCPro and viral intercellular movement have been not demonstrated thus far.

Cell-to-cell movement of plant viruses usually depends on the coordinated action of viral MPs and host proteins [41,42]. Several host proteins have been identified to interact with potyvirid movement-related proteins. A hydrophilic plasma membrane-associated cation-binding protein (PCaP1) is recruited to PD via interaction with P3N-PIPO to promote viral intercellular movement, in cases of turnip mosaic virus (TuMV) and tobacco vein banding mosaic virus [10,24,43]. PCaP1 might function in anchoring P3N-PIPO to PD, or serving actin filaments inside PD to enlarge their SEL [10,43]. Another plasma membrane protein, synaptotagmin A, facilitates the trafficking of TuMV P3N-PIPO through PD [44]. An α-expansin in *N*. *benthamiana* (NbEXPA1) promotes both replication and cell-to-cell movement of TuMV [45].

The subject of chloroplast-virus interplay has been attracting great interest for a long time [46–48]. An increasing number of chloroplast proteins are co-opted by different viruses for replication, movement or/and counteracting host defense response [49–53]. Ribulose 1, 5-bisphosphate carboxylase/oxygenase (Rubisco) catalyzes the first rate-limiting step in CO_2_ fixation in photosynthesis. Rubisco is comprised of eight large subunits (RbCL; 50–55 kDa) and eight small subunits (RbCS; 12–18 kDa) which form a hexadecameric L_8_S_8_ complex [54–56]. RbCL is encoded by chloroplast genome, while RbCS is nucleus-encoded [54]. RbCS interacts with tobamoviral MPs at PD for viral intercellular and long-distance movement [57]. RbCL or RbCS interact with both P3 and P3N-PIPO in cases of several potyviruses [58]. RbCL interacts with HCPro of bean common mosaic virus, and CP of potato virus Y [59,60]. However, the biological relevance of these interactions has not clearly defined.

Previously, we characterized two novel viruses, areca palm necrotic spindle-spot virus (ANSSV) and areca palm ring spot virus (ANRSV), which are clustered into a new genus in the *Potyviridae* family [18,61,62]. Both viruses share a distinct pattern of leader proteases—two copies of HCPro (HCPro1-HCPro2) [63], which prompted us to investigate the functions of HCPro1 and HCPro2 during viral infection. In the present study, we found that HCPro1 is dispensable for viral infection, whereas HCPro2 is indispensable. Besides acting as the viral suppressor of RNA silencing (VSR), HCPro2 participates in viral cell-to-cell movement, which could be functionally complemented by its counterpart from a potyvirus. HCPro2 interacts with both CI and CP *in planta*, and these three viral proteins form the complexes around PD during viral infection. More interestingly, we identified a common host protein, NbRbCS, that likely acts as a scaffold in the formation of a viral complex for viral cell-to-cell movement. Reduced *NbRbCS* gene expression greatly impairs viral intercellular movement and systemic infection for ANRSV and three potyviruses tested.

## Results

### HCPro1 is dispensable for ANRSV infection

**Fig 1:**
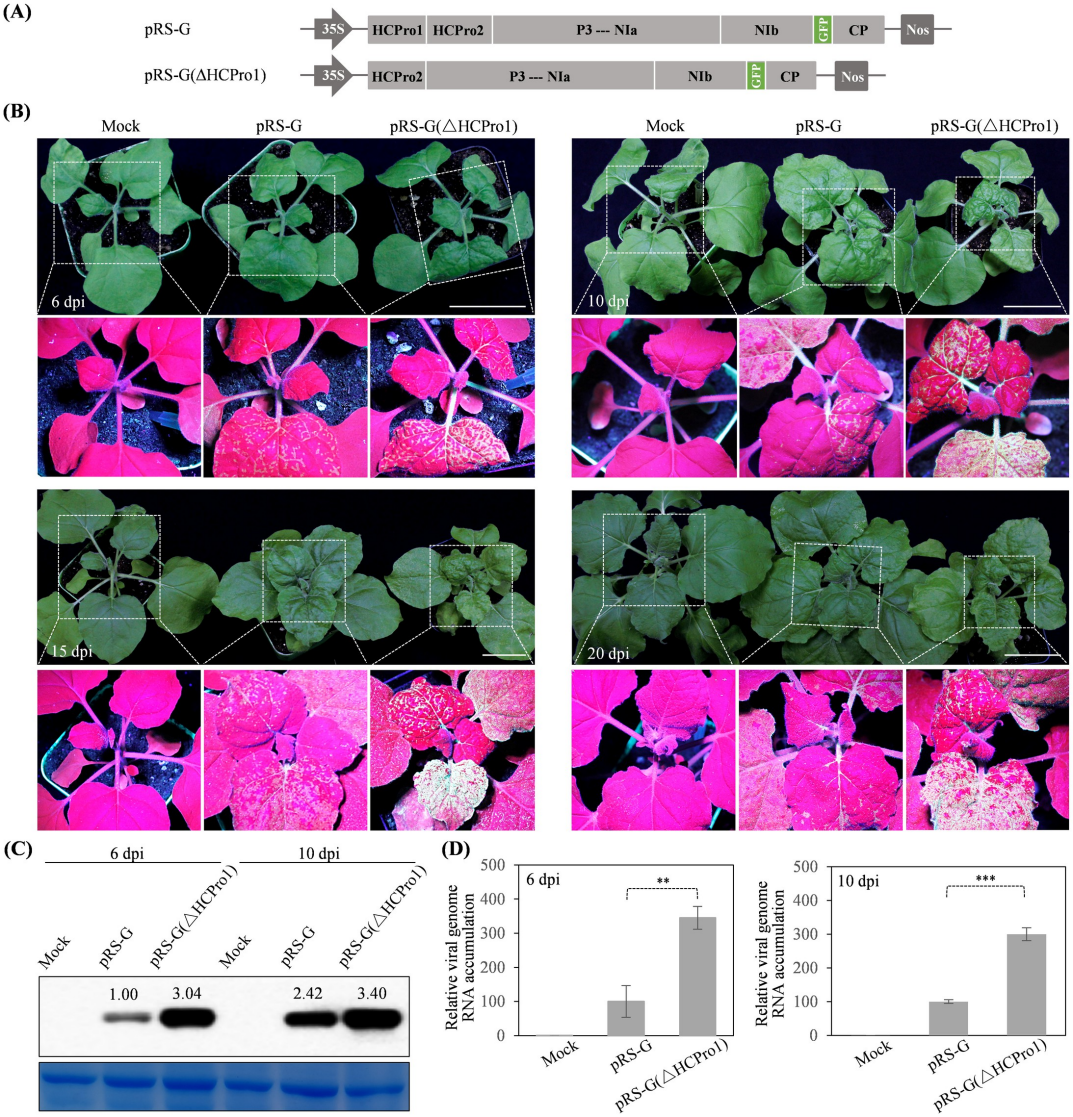
HCPro1 is dispensable for ANRSV infection. (A) Schematic diagrams of pRS-G and pRS-G(ΔHCPro1). The P3—NIa represents the coding region for seven viral factors, including P3, P3N-PIPO, 6K1, CI, 6K2, VPg and NIa-Pro. (B) Infectivity test of pRS-G and pRS-G(ΔHCPro1) in *N*. *benthamiana*. The representative *N*. *benthamiana* plants inoculated with the indicated virus clones were photographed under daylight (upper) and UV light (lower). Mock, empty vector control. Bars, 5 cm. (C) Western blot analysis of GFP accumulation in inoculated *N*. *benthamiana* plants. Total proteins were extracted from top non-inoculated leaves at the indicated time points. The hybridization signal intensity was quantitatively analyzed with ImageJ software [106]. Coomassie blue staining of RbCL was used as a loading control. (D) Real-time RT-qPCR analysis of viral RNA accumulation in inoculated plants. Total RNAs were extracted from top non-inoculated leaves, followed by real-time RT-qPCR analysis. The values represent the mean ± standard deviation (SD) from three independent biological replicates. The average values for pRS-G were designated 100 to normalize the data. Statistically significant differences, determined by an unpaired two-tailed Student’s *t* test, are indicated by asterisks. **, 0.001<*P*<0.01; ***, *P*<0.001.

To examine the function(s) of HCPro1 during ANRSV infection, its coding region was removed from pRS-G to produce pRS-G(ΔHCPro1) (Fig 1A). pRS-G and pRS-G(ΔHCPro1) were each inoculated into ten *N*. *benthamiana* seedlings via agroinfiltration (OD_600_ = 0.5 per clone). At different time points, all plants inoculated with either pRS-G or pRS-G(ΔHCPro1) exhibited dwarfism and leaf rugosity symptoms, as well as obvious GFP signals along veins in top non-inoculated leaves (Fig 1B). Interestingly, more severe symptoms along with stronger fluorescence intensity were observed in plants inoculated with pRS-G(ΔHCPro1) (Fig 1B). Consistently, both GFP and viral genomic RNA accumulated to higher levels in these plants (Fig 1C and 1D). The genomic region, corresponding to partial 5′ UTR (110 nucleotides [nts]), complete HCPro2, and partial P3 (150 nts) for virus progeny derived from pRS-G(ΔHCPro1), was sequenced, and the spontaneous mutations of nucleotide sequence were not observed. Given that the deletion of HCPro1-coding sequence shortens viral genome size, it is uncertain if the enhancement effect on viral infectivity is caused by an alteration of viral genome or a negatively regulatory role exerted by HCPro1 protein. Nevertheless, our data support that HCPro1 is dispensable for ANRSV infection in *N*. *benthamiana* plants.

### HCPro2 functions in viral cell-to-cell movement, which is functionally complemented by its counterpart—HCPro from a potyvirus

**Fig 2:**
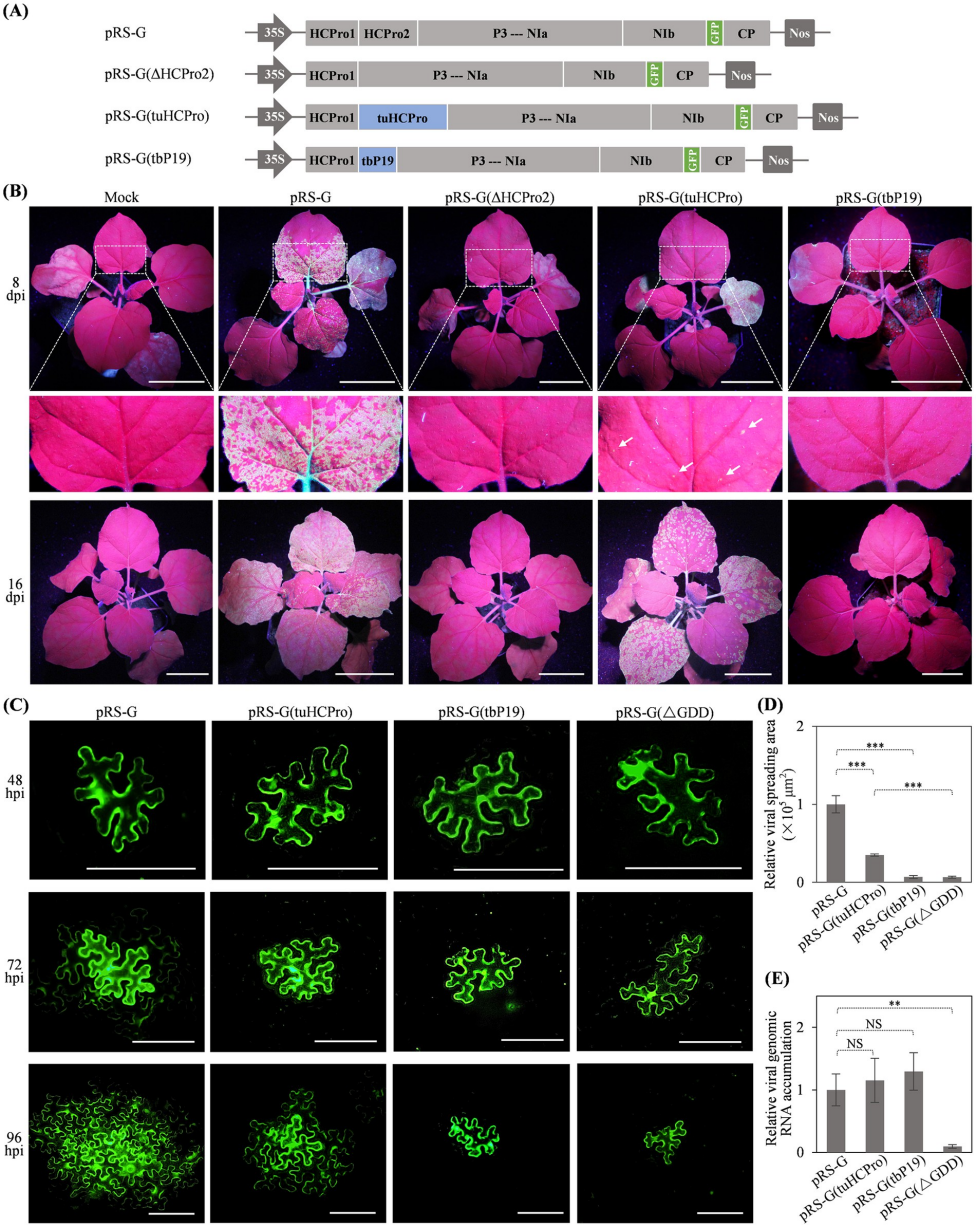
The effects of deletion of HCPro2 or its substitution with different VSRs on viral infectivity. (A) Schematic diagrams of the derivatives of pRS-G. TuMV HCPro and TBSV P19 are represented by tuHCPro and tbP19, respectively. (B) Infectivity test of the derivatives of pRS-G in *N*. *benthamiana*. Representative photographs were taken under UV light at the indicated time points. The close view of leaf regions indicated by dashed boxes is shown. White arrows indicate fluorescence spots. Mock, empty vector control. Bars, 5 cm. (C) Time course observation of viral cell-to-cell movement for the indicated virus clones. Viral intercellular movement was monitored at 48 hpi, 72 hpi, and 96 hpi. Bars, 100 μm. (D) Statistical analysis of the size of viral spreading area at 96 hpi. For each clone, at least 25 infection foci from a total of six plants in three independent experiments were analyzed. The size of infection foci was calculated by ImageJ. The data are presented as the mean ± SD (*n* ≥ 25). The average value for pRS-G was designated 1×10^5^ μm^2^ to normalize the data. Statistically significant differences, determined by an unpaired two-tailed Student’s *t* test, are indicated by asterisks. ***, *P*<0.001. (E) The effects of hybrid virus clones on viral genomic RNA accumulation. Relative viral genomic RNA accumulation was determined by real-time RT-qPCR with a pair of primers RS9200F/RS9350R (S2 Table) targeting viral *CP* region. *N*. *benthamiana* leaves inoculated with the indicated clones (OD_600_ = 0.3 per clone) were sampled at 60 hpi for the assay. Error bars denote the SD from three biological replicates. **, 0.001<*P*<0.01; NS, no significant difference.

To investigate the functions of HCPro2 during viral infection, we deleted HCPro2-coding sequence in pRS-G to generate pRS-G(ΔHCPro2) (Fig 2A). Infectivity test showed that all eight plants inoculated with pRS-G displayed obvious GFP fluorescence in upper non-inoculated leaves at 8 dpi and 16 dpi, whereas those inoculated with pRS-G(ΔHCPro2) did not (Fig 2B). RT-PCR confirmed the absence of viral infection in non-inoculated leaves of all plants treated with pRS-G(ΔHCPro2) (S1 Fig). ANSSV HCPro2 (ssHCPro2) expresses the RSS activity [63]. Thus, we tested the RSS activity of ANRSV HCPro2. For this, we constructed three T-DNA vectors for expressing HA-tagged HCPro1 (HCPro1-HA), HCPro2 (HCPro2-HA) and HCPro1-HCPro2 (HCPro1-HCPro2-HA) of ANRSV, respectively. Each of them, together with a plasmid for expressing GFP reporter [64] were co-inoculated into *N*. *benthamiana* leaves. Co-expression of GFP along with either empty vector or HA-tagged ssHCPro2 (ssHCPro2-HA) was included as the negative and positive controls, respectively. At 60 hours post-inoculation (hpi), the leaf patches co-expressing HCPro2-HA/GFP, HCPro1-HCPro2-HA/GFP or ssHCPro2-HA/GFP displayed strong GFP fluorescence, whereas no obvious fluorescence was observed on the leaf patches co-expressing HCPro1-HA/GFP or negative control (S2A Fig). Consistently, a higher abundance of GFP at both protein and RNA levels was detected in leaf patches co-expressing HCPro2-HA/GFP, HCPro1-HCPro2-HA/GFP or ssHCPro2-HA/GFP (S2B and S2C Fig), indicating that HCPro2 is the VSR of ANRSV.

Both potyvirus-encoded HCPro (the counterpart of ANRSV HCPro2) and tombusvirus-encoded P19 are well-known VSRs [65–67]. To explore additional functions of HCPro2 beyond RSS, we substituted HCPro2 in pRS-G with either TuMV HCPro (tuHCPro) or tomato bushy stunt virus P19 (tbP19) to produce two hybrid clones, pRS-G(tuHCPro) and pRS-G(tbP19) (Fig 2A). *N*. *benthamiana* seedlings (*n* = 10 per clone) were inoculated with them, followed by observations under UV light in every one- or two-day interval for one month. At 8 dpi, obvious fluorescence spots were observed in upper non-inoculated leaves of plants inoculated with pRS-G(tuHCPro). All plants inoculated with either pRS-G(tuHCPro) or wild-type pRS-G displayed the comparable distribution pattern and intensity of fluorescence signals at 13, 16 and 30 dpi (Figs 2B and S3A). In contrast, only three out of 10 plants inoculated with pRS-G(tbP19) showed scattered fluorescence spots in only one non-inoculated leaf at 30 dpi (S3A Fig). For virus progeny derived from three hybrid clones, the genomic sequence, covering partial HCPro1 (200 nts), complete tuHCPro / tbP19, and partial P3 (150 nts) was determined, and the alternations of nt sequences were not identified. Altogether, these results suggested that HCPro2 implements additional function(s) beyond RSS, which can be largely complemented by its counterpart in TuMV.

Further, we examined the performance of hybrid viruses in intercellular movement. Agrobacterial cultures harboring pRS-G, pRS-G(tuHCPro), pRS-G(tbP19) or pRS-G(ΔGDD) (a replication- and movement-null mutant that lacks a strictly-conserved GDD motif in viral RNA polymerase) were highly diluted to 0.0001 of OD_600_, and infiltrated into *N*. *benthamiana* leaves. Single cells emitting GFP fluorescence, representing primarily-transfected cells, were observed for all clones at 48 hpi and 60 hpi (Figs 2C and S3B). Clear viral spreading from primarily-transfected to peripheral cells started at 72 hpi for pRS-G, and 84 hpi for pRS-G(tuHCPro) (Figs 2C and S3B). Thus, replacement of HCPro2 with tuHCPro partially inhibited viral intercellular movement (Figs 2D and S3C). In contrast, pRS-G(tbP19), similar to pRS-G(ΔGDD), was deficient in cell-to-cell movement (Figs 2C and S3B). Moreover, we assessed the performance of hybrid viruses in viral genomic RNA accumulation. *N*. *benthamiana* leaves inoculated with these clones were used in real-time RT-qPCR to measure virus accumulation at 60 hpi as viral intercellular movement did not occur at this time point (S3B Fig). As shown in Fig 2E, no significant difference was found between wild-type pRS-G and each of hybrid clones. Conclusively, HCPro2 also functions in viral cell-to-cell movement.

### HCPro2 forms PD-localized punctate inclusions in virus-infected cells

**Fig 3:**
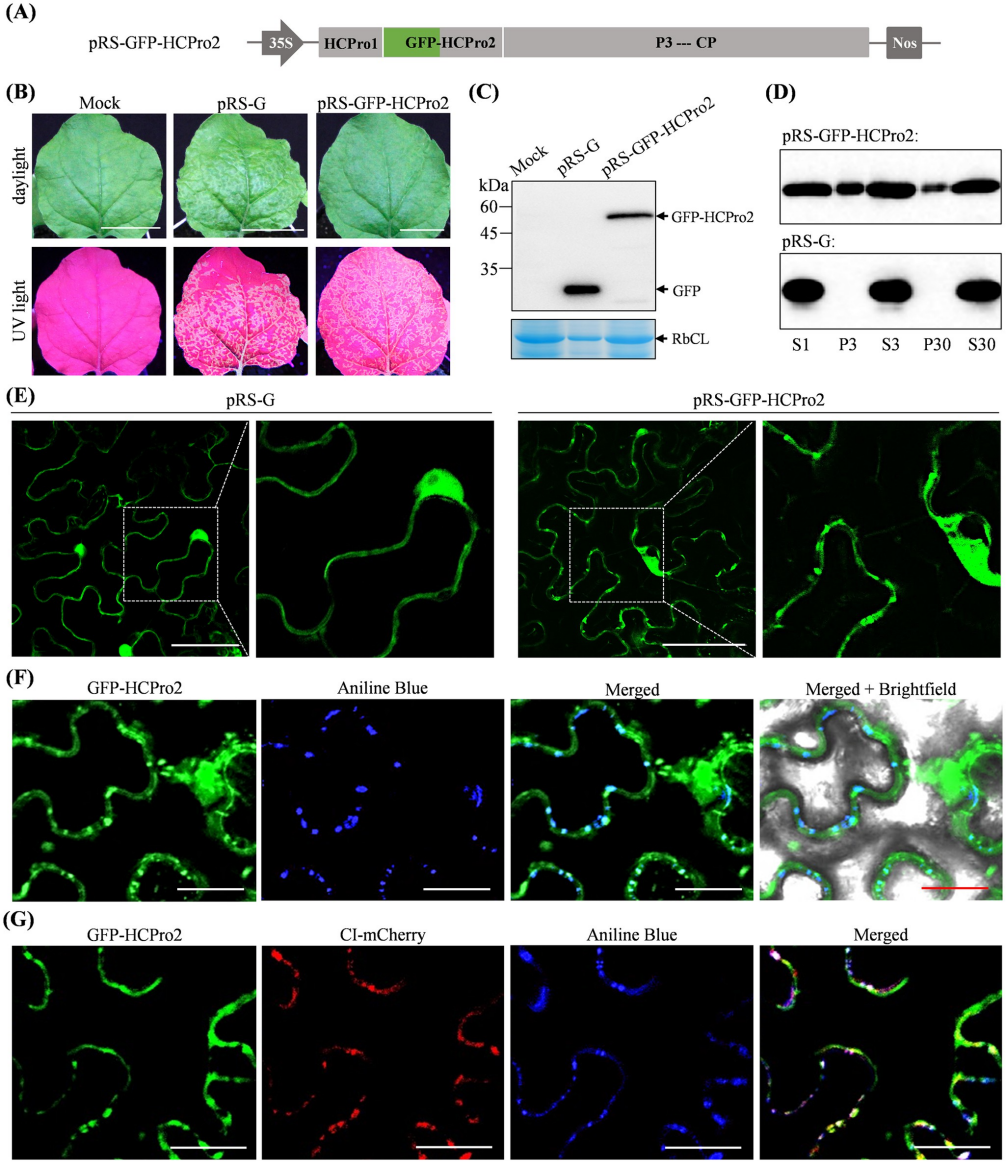
Cellular compartment distribution and subcellular localization of HCPro2 in virus-infected cells. (A) Schematic diagram of pRS-GFP-HCPro2. For the clone, the complete GFP-coding sequence was fused at the N-terminus of HCPro2. (B) Infectivity test of pRS-GFP-HCPro2 in *N*. *benthamiana*. The upper non-inoculated leaf was photographed under daylight and UV light at 8 dpi. Mock, empty vector control. Bars, 2.5 cm. (C) Immunoblot detection of GFP-HCPro2 accumulation. The upper non-inoculated leaves of *N*. *benthamiana* plants infiltrated with pRS-GFP-HCPro2 or pRS-G were assayed by Western blot at 8 dpi. Coomassie blue staining of RbCL was used as a loading control. (D) Subcellular fractionation coupled with immunoblot detection of GFP-HCPro2. The upper non-inoculated leaves of *N*. *benthamiana* plants infiltrated with pRS-GFP-HCPro2 or pRS-G were collected at 8 dpi for subcellular fractionation assay. The resulting fractions were subjected to immunoblot detection by using anti-GFP anti-body. S1, the supernatant following centrifugation of crude homogenate at 1000 *g*; S3 and P3, the corresponding supernatant and pellet following the centrifugation of S1 at 3700 *g*; S30 and P30, the corresponding supernatant and pellet following the centrifugation of S3 at 30000 *g*. (E) Subcellular localization of GFP-HCPro2 in virus-infected cells. *N*. *benthamiana* leaves were inoculated with pRS-GFP-HCPro2 or pRS-G, followed by confocal microscopy observation at 72 hpi. The regions indicated by dashed boxes are enlarged. Bars, 50 μm. (F) Subcellular co-localization of GFP-HCPro2 and the callose at PD. At 72 hpi, the inoculated leaves with pRS-GFP-HCPro2 were stained with aniline blue, followed by confocal microscopy observation. Bars, 25 μm. (G) Co-localization of HCPro2 and CI at PD in virus-infected cells. *N*. *benthamiana* leaves were co-inoculated with pRS-GFP-HCPro2 together with a construct for expressing CI-mCherry (final OD_600_ = 0.2 per clone), followed by staining with aniline blue at 72 hpi and confocal microscopy observation. Bars, 25 μm.

To investigate the cellular compartment distribution of HCPro2 in virus-infected cells, we fused a GFP-coding sequence at the beginning of HCPro2 in pRS to obtain pRS-GFP-HCPro2 (Fig 3A). Infectivity test showed all inoculated plants (*n* = 10) exhibited chlorosis and obvious fluorescence signals along veins in upper non-inoculated leaves (Fig 3B), indicating that the recombinant clone is viable. The fused GFP-HCPro2 (61.21 kDa) was detected (Fig 3C). Virus-infected leaf tissues were sampled for subcellular fractionation assay. Immunoblot analysis revealed that GFP-HCPro2 was present in different fractions with a varied degree, including nuclei-chloroplast-cell wall fraction (P3), membranous fraction (P30), and cytoplasmic fraction (S30) (Fig 3D). As a control, free GFP produced in pRS-G sample was mainly present in S30 fraction (Fig 3D).

Next, we examined the subcellular localization pattern of HCPro2 in virus-infected cells. *N*. *benthamiana* leaves infiltrated with either pRS-GFP-HCPro2 or pRS-G (OD_600_ = 0.1) at 72 hpi were subjected to confocal microscopy observation. Both free GFP in pRS-G sample and GFP-HCPro2 in pRS-GFP-HCPro2 were observed to be diffused into cytoplasm and nucleus (Fig 3E). Differently, GFP-HCPro2 was also aggregated in punctate structures, and the distribution pattern resembles that of PD-localized markers (Fig 3E). To test this idea, leaf samples of pRS-GFP-HCPro2 were stained with aniline blue, which reacts with callose deposited at PD necks. As expected, about 70% of GFP-HCPro2 punctate (82 out of 120 punctate observed) colocalized with aniline blue-stained callose (Fig 3F). CI is a PD-localized viral protein in viral infection [20]. We produced a T-DNA construct for the expression of mCherry-fused CI (CI-mCherry). The construct together with pRS-GFP-HCPro2 were co-inoculated into *N*. *benthamiana* leaves. The majority of GFP-HCPro2 punctate structures (112 out of 150 inclusions observed) were overlapped with both CI-mCherry inclusions and aniline blue-stained callose structures at 72 hpi (Fig 3G). Taken together, HCPro2 is distributed into different cellular compartments, and in particular forms PD-localized inclusions in virus-infected cells, providing an important clue on the involvement of HCPro2 in viral cell-to-cell movement.

### Purification and identification of viral and host proteins that physically associate with HCPro2 in the context of viral infection

**Fig 4:**
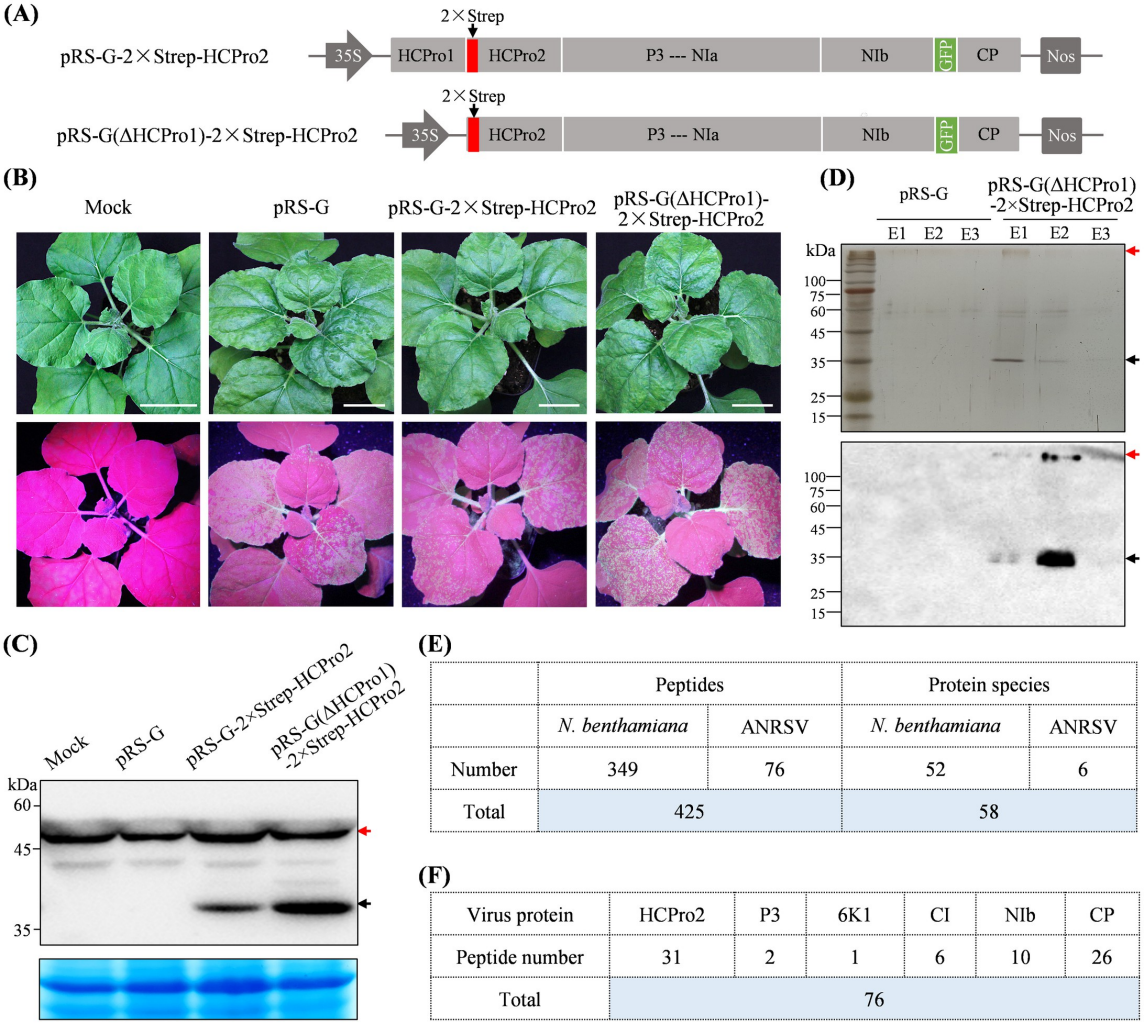
Purification and identification of viral and host proteins that associate with HCPro2 during ANRSV infection. (A) Schematic diagrams of pRS-G-2×Strep-HCPro2 and pRS-G(ΔHCPro1)-2×Strep-HCPro2. (B) Infectivity test of the indicated clones in *N*. *benthamiana*. The representative plants were photographed at 12 dpi. Bars, 2.5 cm. (C) Immunoblot detection of 2×Strep-HCPro2 in upper non-inoculated leaves at 12 dpi. Coomassie blue staining of RbCL was used as a loading control. The bands corresponding to the expected size of 2×Strep-HCPro2 (37.57 kDa) are indicated by black arrow. The red arrow indicates unspecific bands. (D) SDS-PAGE analysis and immunoblot detection of co-purified proteins with 2×Strep-HCPro2. The upper non-inoculated leaves of plants infiltrated with pRS-G (as the parallel control) or pRS-G(ΔHCPro1)-2×Strep-HCPro2 were collected at 12 dpi for affinity-purification with streptavidin. Elution fractions (E1-E3) were used for SDS-PAGE with silver staining (upper panel) and immunoblot detection (lower panel). The black arrow indicates putative bands corresponding to 2×Strep-HCPro2. The bands (indicated by red arrows) likely represent a HCPro2-containing complex with a high-molecular-mass. (E) LC-MS/MS identification of co-purified products with 2×Strep-HCPro2. The co-purified products from both pRS-G and pRS-G(ΔHCPro1)-2×Strep-HCPro2 samples were analysed by LC-MS/MS. The protein species, uniquely identified from co-purified products with 2×Strep-HCPro2, together their corresponding peptides were summarized. (F) Summary of viral proteins co-purified with 2×Strep-HCPro2.

To get insight into the role of HCPro2 in viral intercellular movement, a twin-Strep sequence (2×Strep) was fused with the first nucleotide of HCPro2 in pRS-G (Fig 4A) to purify viral and host proteins that physically associate with HCPro2 in the context of viral infection. Infectivity test showed that pRS-G-2×Strep-HCPro2 is viable, but much weaker than wild-type pRS-G (Figs 4B and S4). The fused 2×Strep-HCPro2 (37.57 kDa) was detected from upper non-inoculated leaves (Fig 4C). Unfortunately, streptavidin purification failed to enrich 2×Strep-HCPro2 along with its associating proteins via SDS-PAGE analysis and immunoblot detection. Considered that HCPro1 deletion significantly increases both viral RNA load and protein expression (Fig 1C and 1D), we used pRS-G(ΔHCPro1) instead to fuse the 2×Strep with HCPro2, and obtained pRS-G(ΔHCPro1)-2×Strep-HCPro2 (Fig 4A). Infectivity test showed that the clone was more aggressive in both virus-triggered symptoms and systemic spreading (Fig 4B). A significantly higher abundance of 2×Strep-HCPro2 and viral RNA accumulation was detected (Figs 4C and S4). The upper non-inoculated leaves of plants were subjected to affinity purification. SDS-PAGE analysis revealed the presence of a putative band corresponding to the expected size of 2×Strep-HCPro2 and several other bands in co-purified products, whereas these bands were absent in the parallel control pRS-G (Fig 4D, upper panel). The presence of 2×Strep-HCPro2 was verified by immunoblotting (Fig 4D, lower panel). The affinity-purified products from both samples were analyzed by liquid chromatography tandem mass spectrometry (LC-MS/MS). A total of 58 protein species, including six viral proteins (HCPro2, P3, 6K1, CI, NIb, and CP) and 52 host proteins, were uniquely identified in co-purified products with 2×Strep-HCPro2 (Fig 4E and 4F, and S1 Table).

### HCPro2 interacts with CI and CP *in planta*

**Fig 5:**
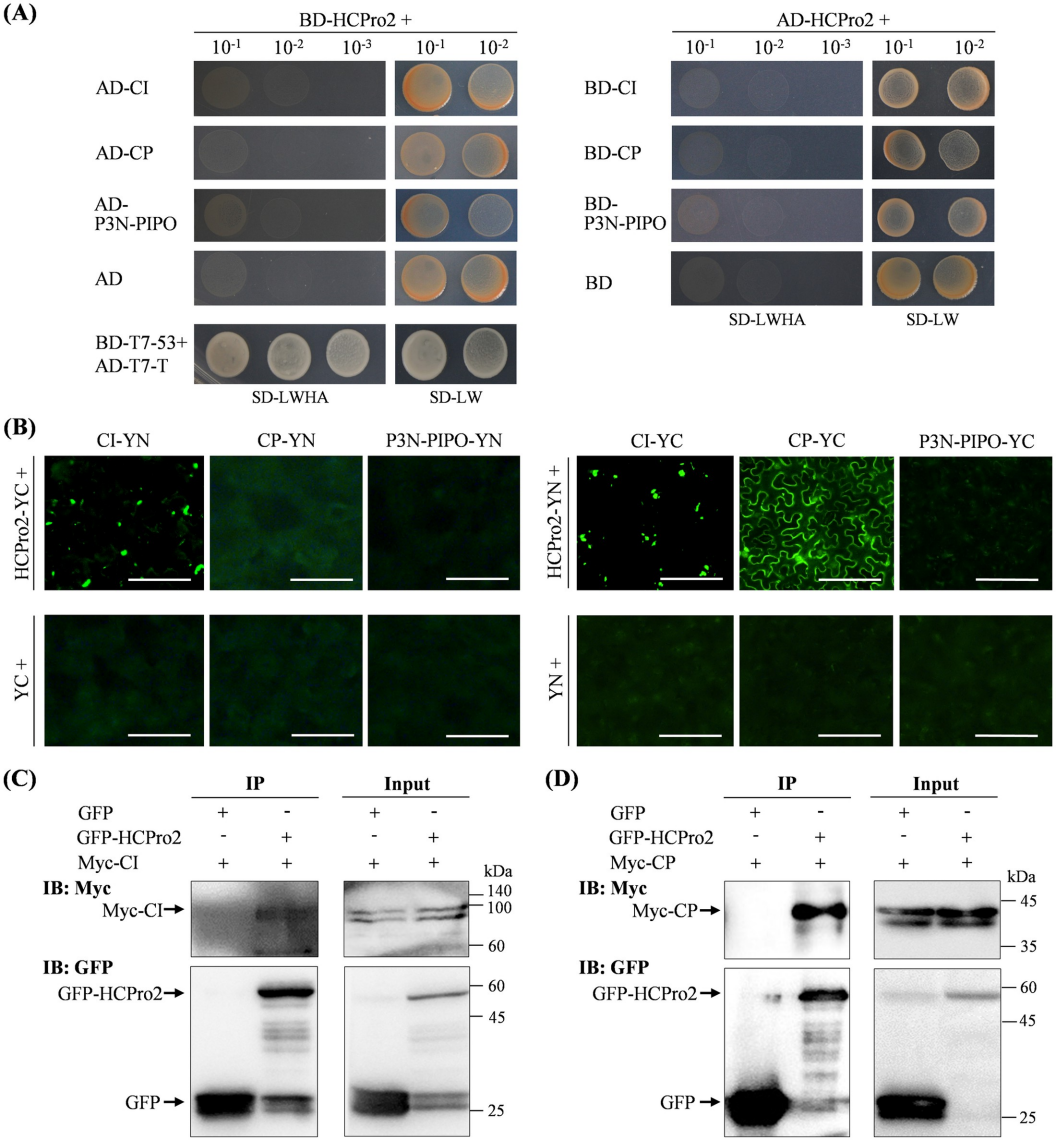
HCPro2 interacts with CI and CP *in planta*. (A) Y2H tests the interactions of HCPro2 with P3N-PIPO, CI and CP. The coding sequences of HCPro2, P3N-PIPO, CI and CP were cloned into pGBKT7-DEST or pGADT7-DEST for the expression of these proteins fused with GAL4 BD or AD domain. Yeast competent cells (Y2H Gold) were co-transformed to express the indicated pairs of proteins. The transformed cells were subjected to 10-fold serial dilutions and plated on the SD/-Trp/-Leu and SD/-Trp/-Leu/-His/-Ade mediums. The plates were cultured at 28°C for four to six days before photographing. Co-transformation of a pair of plasmids for simultaneous expression of AD-T7-T and BD-T7-53 was included as the positive control. (B) BiFC tests the interactions of HCPro2 with CI, CP and P3N-PIPO. The coding sequences of HCPro2, CI, CP and P3N-PIPO were individually engineered into pEarleyGate201-YN and pEarleyGate202-YC for the expression of these proteins fused with the YN or YC part of YFP. *N*. *benthamiana* leaves were co-inoculated for the expression of the indicated pairs of proteins. YFP signals (shown in green) were observed by fluorescence microscope at 72 hpi. Bars, 50 μm. (C, D) Co-IP tests the interactions of HCPro2 with CI and CP. The inoculated leaves of *N*. *benthamiana* plants for co-expression of GFP-HCPro2 / Myc-CI (C) or GFP-HCPro2 / Myc-CP (D) were sampled at 72 hpi for Co-IP assays using GFP-Trap Agarose. Total protein extracts prior to (Input) and after immunoprecipitation (IP) were analyzed by immunoblotting using anti-Myc and anti-GFP antibodies.

Both CI and CP (potyvirid movement-related proteins) are co-purified with HCPro2, prompting us to envisage that HCPro2 might regulate viral intercellular movement via interactions with CI and CP. Thus, we examined the interactions of HCPro2 with three viral movement-related factors (CI, CP, and P3N-PIPO) by using yeast two-hybrid (Y2H). Their coding sequences were cloned into pGBKT7-DEST or pGADT7-DEST. Co-transformation of yeast cells did not detect the interaction between BD-HCPro2 and AD-CI, AD-CP or AD-P3N-PIPO (Fig 5A). A consistent result was obtained when co-expressing AD-HCPro2 and BD-CI, BD-CP or BD-P3N-PIPO (Figs 5A and S5). HCPro2 did not interact with the remaining viral factors in Y2H either (S6 Fig). As well, the interactions of HCPro2 with CI, CP, and P3N-PIPO were not identified when tested by membrane yeast two hybrid (MYTH) (S7 Fig).

Next, we examined whether HCPro2 interacts with CI, CP and P3N-PIPO *in planta* using bimolecular fluorescence complementation (BiFC). Their coding sequences were individually engineered into both pEarleyGate201-YN and pEarleyGate202-YC. HCPro2-YC along with P3N-PIPO-YN, CI-YN or CP-YN were co-expressed in *N*. *benthamiana* leaves. Obvious fluorescence signals with punctate distribution were observed for the co-expression of HCPro2-YC and CI-YN (Fig 5B, left panel), indicating that HCPro2 interacts with CI *in planta*. A consistent result was obtained when using a combination of HCPro2-YN and CI-YC for the test (Fig 5B, right panel). In addition, we observed strong fluorescence signals in leaf samples co-expressing HCPro2-YN and CP-YC (Fig 5B). In contrast, no interaction was detected between HCPro2 and P3N-PIPO (Fig 5B). Further, the interactions of HCPro2 with CI and CP were tested by co-immunoprecipitation (Co-IP). For this, we developed a series of T-DNA constructs for transient expression of free GFP, GFP-tagged HCPro2 (GFP-HCPro2), and 4×Myc-tagged CI (Myc-CI) and CP (Myc-CP). GFP-HCPro2 was co-expressed with Myc-CI or Myc-CP in *N*. *benthamiana* leaves. Co-expression of GFP and Myc-CI or Myc-CP was included as the parallel controls. Total proteins were subjected to co-immunoprecipitation with GFP-Trap Agarose. Immunoblot analysis showed that both CI and CP were co-immunoprecipitated with GFP-HCPro2, but not with free GFP in control groups (Fig 5C and 5D).

### HCPro2, CI and CP form the complexes at PD in viral infection

**Fig 6:**
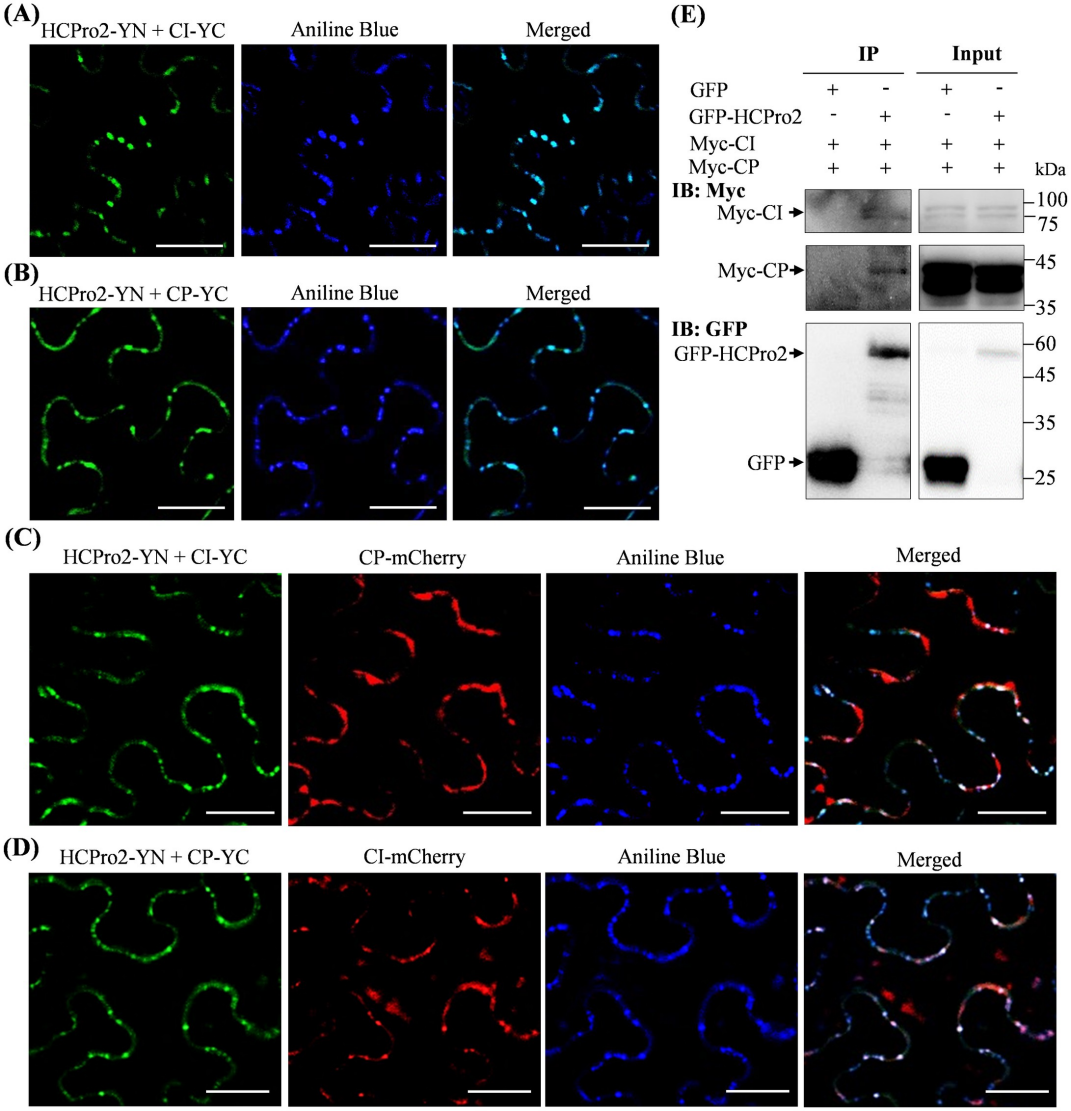
HCPro2, CI and CP form the complexes at PD in viral infection. (A) HCPro2 interacts with CI at PD. *N*. *benthamiana* leaves were co-inoculated with two constructs corresponding to HCPro2-YN and CI-YC together with viral clone–pRS (final OD_600_ = 0.2 per clone), followed by staining with aniline blue at 72 hpi and observation by confocal microscopy. Bars, 25 μm. (B) HCPro2 interacts with CP at PD. *N*. *benthamiana* leaves were co-inoculated with two constructs corresponding to HCPro2-YN and CP-YC together with pRS (final OD_600_ = 0.2 per clone), followed by staining with aniline blue at 72 hpi and observation by confocal microscopy. Bars, 25 μm. (C) Confocal microscopy observation of *N*. *benthamiana* leaves co-expressing HCPro2-YN, CI-YC and CP-mCherry in viral infection. *N*. *benthamiana* leaves were co-inoculated with three constructs for simultaneous expression of HCPro2-YN, CI-YC, and CP-mCherry together with pRS (final OD_600_ = 0.2 per clone), followed by staining with aniline blue at 72 hpi and observation by confocal microscopy. Bars, 25 μm. (D) Confocal microscopy observation of *N*. *benthamiana* leaves co-expressing HCPro2-YN, CP-YC and CI-mCherry in viral infection. *N*. *benthamiana* leaves were co-inoculated with three constructs for simultaneous expression of HCPro2-YN, CP-YC and CI-mCherry along with pRS (final OD_600_ = 0.2 per clone), followed by staining with aniline blue at 72 hpi and observation by confocal microscopy. Bars, 25 μm. (E) Both CI and CP were coimmunoprecipitated with GFP-HCPro2 in viral infection. *N*. *benthamiana* leaves are co-inoculated with two constructs for simultaneous expression of Myc-CI and Myc-CP along with viral clone pRS-GFP-HCPro2. At 72 hpi, total proteins were extracted for Co-IP assay using GFP-Trap Agarose. Total protein extracts prior to (Input) and after immunoprecipitation (IP) were immuno-detected using anti-Myc and anti-GFP polyclonal antibodies.

We further investigated whether the interactions of HCPro2 with CI and CP occur at PD in viral infection. For this, two constructs for co-expression of HCPro2-YN and CI-YC, along with pRS, were co-inoculated into *N*. *benthamiana* leaves. At 72 hpi, obvious fluorescence signals with punctate structures, an indication of the interaction between HCPro2 and CI, were observed. Statistically, 130 out of 150 inclusion observed (approximately 87%) were overlapped with aniline blue-strained callose structures at PD (Fig 6A). Similarly, HCPro2-YN and CP-YC interact to form punctate structures, and a large number of them (126 out of 150 inclusions observed) localized at PD either (Fig 6B). Next, we investigated whether the three viral factors form the complexes at PD in viral infection. Three constructs for simultaneous expression of HCPro2-YN, CI-YC, and CP-mCherry, together with pRS, were inoculated into *N*. *benthamiana* leaves. At 72 hpi, approximately 65% of the punctate structures (85 out of 130 inclusions observed), resulting from the interaction between HCPro2 and CI, overlapped with the structures formed by CP-mCherry at PD (Fig 6C). When HCPro2-YN, CP-YC and CI-mCherry were co-expressed, 95 out of 120 punctate inclusions observed (an indication of the interaction between HCPro2 and CP) colocalized with CI-mCherry structures at PD (Fig 6D). The above results indicate that HCPro2, CI, and CP likely form the complexes at PD. To further prove the existence of HCPro2-CI-CP complex, a Co-IP assay was performed. Two constructs for expressing Myc-CI and Myc-CP, together with pRS-GFP-HCPro2 or pRS-G (as the parallel control), were co-inoculated into *N*. *benthamiana* leaves. Total proteins were subjected to co-immunoprecipitation with GFP-Trap Agarose. Immunoblot analysis showed that both CI and CP were coimmunoprecipitated with GFP-HCPro2 (Fig 6E). Conclusively, the three viral proteins (HCPro2, CI and CP) form an interactive complex at PD in viral infection.

### A common host protein (NbRbCS) facilitates the interactions of HCPro2 with CI or CP, and CI with CP

**Fig 7:**
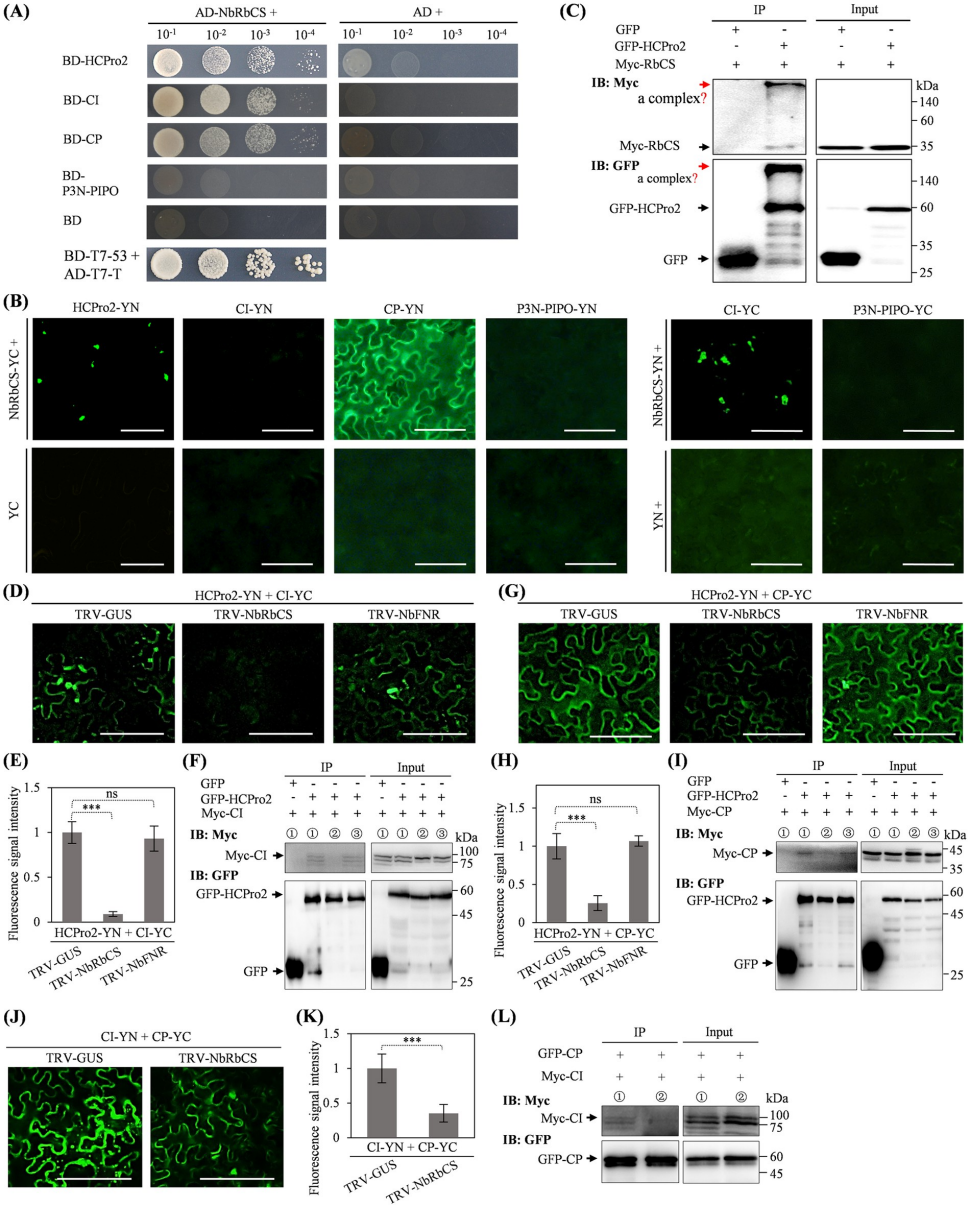
A common host protein—NbRbCS mediates the interactions of HCPro2-CI, HCPro2-CP and CI-CP. (A) Y2H tests the interactions of NbRbCS with HCPro2, CI, CP and P3N-PIPO. Co-transformation of a pair of plasmids for the expression of AD-T7-T and BD-T7-53 was included as the positive control. (B) BiFC tests the interactions of NbRbCS with HCPro2, CI, CP, and P3N-PIPO. *N*. *benthamiana* leaves were co-inoculated for expressing the indicated pair of proteins (final OD_600_ = 0.2 per plasmid). The fluorescence signals (shown in green) were observed by a fluorescence microscope at 72 hpi. Bars, 50 μm. The co-expression of YC or YN along with an indicated protein was included as the negative control. (C) Co-IP tests the interaction of NbRbCS with HCPro2. *N*. *benthamiana* leaves for co-expression of Myc-NbRbCS and GFP-HCPro2 or GFP (final OD_600_ = 0.3 per plasmid) were sampled at 72 hpi for Co-IP assay using GFP-Trap Agarose. The bands indicated by red arrows likely represent a putative complex that contains GFP-HCPro2 and Myc-NbRbCS. (D, G, J) BiFC assays test the interactions of HCPro2-CI, HCPro2-CP, and CI-CP in *NbRbCS*- and *NbFNR*-silenced *N*. *benthamiana* plants. At 12 dpi, the upper fully-expanded leaves were co-inoculated for co-expression of HCPro2-YN / CI-YC (D), HCPro2-YN / CP-YC (G), or CI-YN / CP-YC (J). The OD_600_ value for each plasmid is finally adjusted to 0.2. The samples were observed by a fluorescence microscopy at 60 hpi (G) or 72 hpi (D, J). Bars, 100 μm (D, G) or 50 μm (J). (E, H, K) Statistical analysis of fluorescence signal intensity. The fluorescence signal intensity for HCPro2-YN / CI-YC (E), HCPro2-YN / CP-YC (H), or CI-YN / CP-YC (K), was quantified by ImageJ. At least 20 scans per treatment from three independent experiments were analyzed. Data are presented as the mean ± SD (*n* ≥ 20). ***, *P*<0.001; ns, no significant difference. (F, I, L) Co-IP assays test the interactions of HCPro2-CI, HCPro2-CP and CI-CP in *NbRbCS*- and *NbFNR*-silenced plants. *N*. *benthamiana* plants were pre-inoculated with the indicated TRV-based constructs. Twelve days later, the upper fully-expanded leaves were subjected to co-expression of GFP / GFP-HCPro2 and Myc-CI (F), GFP / GFP-HCPro2 and Myc-CP (I), or GFP-CP and Myc-CI (L) (final OD_600_ = 0.2 per plasmid). The leaf samples were collected at 60 hpi (I) or 72 hpi (F, L) for Co-IP assays using GFP-Trap Agarose. Total protein extracts prior to (Input) and after immunoprecipitation (IP) were analyzed by immunoblotting using anti-Myc and anti-GFP antibodies. The numbers 1, 2 and 3 in circle indicate *N*. *benthamiana* plants pre-inoculated with TRV-GUS, TRV-NbRbCS and TRV-NbFNR, respectively.

Given that HCPro2 interacts with CI and CP by BiFC and Co-IP but not by Y2H and MYTH (Figs 5 and S7), we proposed that one or more host proteins mediate these interactions. To test this hypothesis, Y2H was employed to screen the interactions between HCPro2 and its associating host proteins. The candidate proteins identified by LC-MS/MS with the score above 25 (S1 Table) were selected. The results revealed a strong interaction between HCPro2 and NbRbCS (Fig 7A). This interaction was verified by BiFC and Co-IP (Fig 7B and 7C). Both N-terminal region (N2) and C-terminal cysteine protease region (D2) of HCPro2 interacts with NbRbCS (S8 Fig). Subsequently, we tested whether NbRbCS interacts with CI and CP. Y2H assays showed that AD-NbRbCS interacts with both BD-CI and BD-CP (Fig 7A). The interactions were verified by BiFC (Fig 7B). NbRbCS does not interact with P3N-PIPO, assayed by either Y2H or BiFC (Fig 7A and 7B). In addition, we performed Y2H assays to examine the interactions of NbRbCS with the remaining viral factors (HCPro1, P3, 6K1, 6K2, VPg, NIa-Pro and NIb). Remarkably, the strong interactions of NbRbCS with HCPro1, P3, VPg, and NIb were detected (S9 Fig).

To further illustrate the role of NbRbCS in mediating these interactions, we employed tobacco rattle virus (TRV)-based virus-induced gene silencing (VIGS) to knockdown *NbRbCS* in *N*. *benthamiana*. At 12 dpi, the plants inoculated with TRV-NbRbCS exhibited abnormal development phenotype such as dwarfism in size and foliar yellowing, which was absent in control plants (TRV-GUS) (S10A Fig). Real-time RT-qPCR confirmed that *NbRbCS* mRNA transcripts are significantly reduced in plants inoculated with TRV-NbRbCS (S10B Fig). The upper leaves of *NbRbCS*-silenced and control plants were subjected to co-expression of HCPro2-YN and CI-YC. At 72 hpi, strong fluorescence signals resulting from the interaction between HCPro2 and CI were monitored in control samples, whereas the signals were nearly undetectable in *NbRbCS*-silenced plants (Fig 7D and 7E). Both HCPro2-YN and CI-YC in *NbRbCS*-silenced plants accumulate at a comparable level with those in control plants (S11A Fig). The abundance of RbCL is controlled by its interaction with RbCS to form L_8_S_8_ complex [68]. Supporting this notion, we observed that NbRbCL accumulated less in *NbRbCS*-silenced plants (S11A Fig, lower panel). Co-IP confirmed that the interaction of HCPro2 with CI was greatly weakened in *NbRbCS*-silenced plants (Fig 7F). Similarly, the interaction between HCPro2 and CP was significantly attenuated in *NbRbCS*-silenced plants when tested by BiFC and Co-IP assays (Figs 7G–7I and S11B). Silencing of *NbRbCS* destroys photosynthetic pathway, leading to abnormal physiological phenotype. To discriminate whether the effects of knocking down *NbRbCS* on the interactions of HCPro2 with CI and CP are caused by the deficiency-of-photosynthesis, we silenced another key gene—Ferredoxin-NADP reductase (*FNR*) in photosynthetic pathway. As expected, silencing of *NbFNR* leads to the similar abnormalities as observed in *NbRbCS*-silenced plants (S10A and S10C Fig). Both BiFC and Co-IP revealed that silencing of *NbFNR* did not affect the interactions of HCPro2 with CI and CP, in contrast to those observed in *NbRbCS*-silenced plants (Figs 7D–7I and S11A and S11B).

Potyvirid CP or virion binds with CI-forming conical structures to aid viral cell-to-cell movement, whereas the interaction of CI and CP was detected *in planta* in most cases. Since NbRbCS interacts with both CI and CP, we proposed that NbRbCS mediates the interaction between CI and CP either. To test this hypothesis, CI-YN and CP-YC were co-expressed in *NbRbCS*-silenced leaves. At 72 hpi, strong fluorescence signals resulting from CI-CP interaction were observed in control samples, whereas this interaction was significantly compromised in *NbRbCS*-silenced plants (Figs 7J and 7K and S11C). Co-IP further confirmed the above results (Fig 7L). Notably, the removal of chloroplast transit peptide (CTP) in NbRbCS, preventing its entering into chloroplast, does not affect its interactions with CI and CP, indicating that the portion of NbRbCS in cytoplasm is sufficient to mediate these interactions (S12 Fig). Altogether, NbRbCS acts as a common host protein to mediate the interactions of HCPro2 with CI or CP, and CI with CP.

### Interactions of NbRbCS with HCPro2, CI and CP occur at PD in viral infection

**Fig 8:**
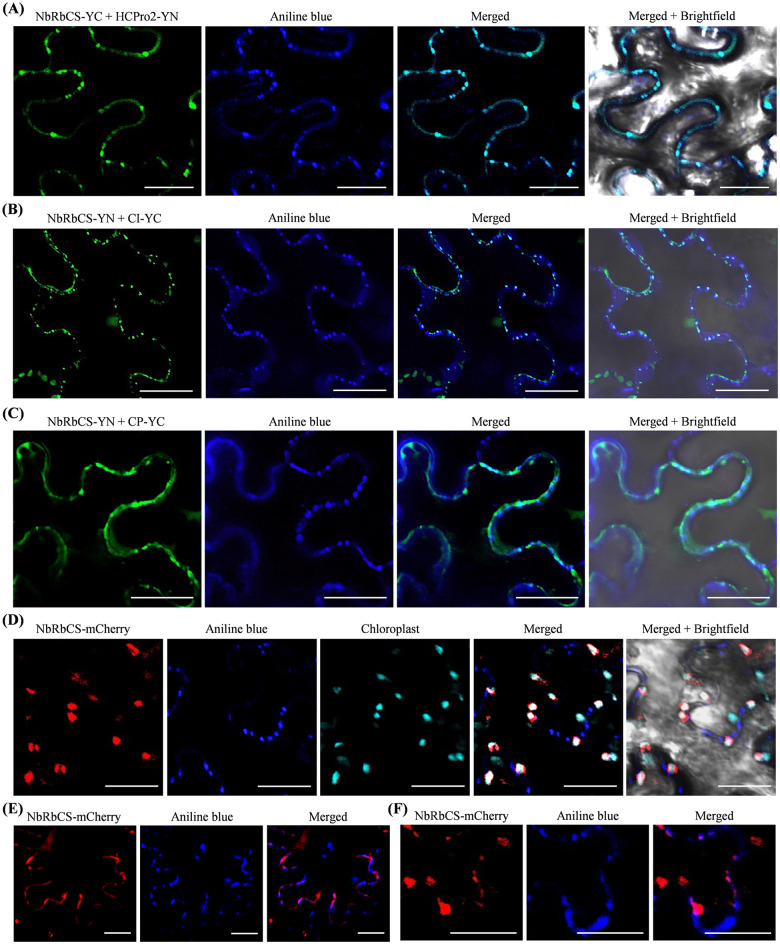
NbRbCS interacts with HCPro2, CI and CP at PD during viral infection. (A-C) The interactions of NbRbCS with HCPro2, CI, and CP occur at PD in viral infection. A pair of constructs for co-expression of NbRbCS-YC / HCPro2-YN (A), NbRbCS-YN / CI-YC (B), or NbRbCS-YN / CP-YC (C), together with viral clone—pRS were co-inoculated into *N*. *benthamiana* leaves (final OD_600_ = 0.2 per clone). The inoculated leaves were stained with aniline blue at 72 hpi, followed by confocal microscopy observation. Bars, 25 μm. (D) NbRbCS-mCherry targets chloroplast when expressed alone *in planta*. *N*. *benthamiana* leaves were inoculated with the construct of NbRbCS-mCherry (OD_600_ = 0.2), followed by aniline blue staining at 72 hpi and confocal microscopy observation. Bars, 25 μm. (E, F) Subcellular localization of NbRbCS-mCherry at PD in viral infection. *N*. *benthamiana* leaves were co-expressed with NbRbCS-mCherry and viral clone (pRS) (OD_600_ = 0.2 per clone), followed by aniline blue staining at 72 hpi and confocal microscopy observation. A close-view of co-localization of NbRbCS-mCherry with aniline blue-stained callose at PD is shown in panel (F). Bars, 25 μm.

As illustrated above, HCPro2, CI, and CP form the complexes at PD during viral infection, and the interactions among them are mediated by a common host protein—NbRbCS. These prompted us to speculate that NbRbCS interacts with the three viral factors at PD during viral infection. Each pair of T-DNA constructs for co-expression of RbCS and HCPro2, CI or CP, together with viral clone pRS, were co-inoculated into *N*. *benthamiana* leaves, followed by aniline blue staining at 72 hpi. Confocal microscopy revealed that the interactions of NbRbCS with HCPro2, CI, and CP consistently form punctate inclusions, which are largely overlapped with aniline blue-stained callose structures at PD (Fig 8A–8C). Statistically, 90 out of 115 inclusions observed for NbRbCS-HCPro2, 95 out of 110 for NbRbCS-CI, and 40 out of 65 for NbRbCS-CP co-localized with callose structures at PD. RbCS is a nucleus-encoded protein and transported into chloroplast via its N-terminal transit peptide. Hence, we examined whether NbRbCS was recruited to PD during viral infection. For this, we generated a construct for expressing a mCherry-tagged NbRbCS (NbRbCS-mCherry). As shown in Fig 8D, NbRbCS-mCherry, when expressed alone, exactly localized at chloroplast, but not at PD at all. Intriguingly, when NbRbCS-mCherry was co-expressed along with viral clone pRS, it was diffused in the cytoplasm or formed punctate inclusions (Fig 8E and 8F). Statistically, approximately 41% of punctate structures (35 out of 110 inclusions observed) were overlapped with alanine blue-stained callose at PD (Fig 8E and 8F).

### Knockdown of *NbRbCS* significantly attenuates viral cell-to-cell movement and systemic infection for ANRSV and other three tested viruses in *Potyvirus* genus

**Fig 9:**
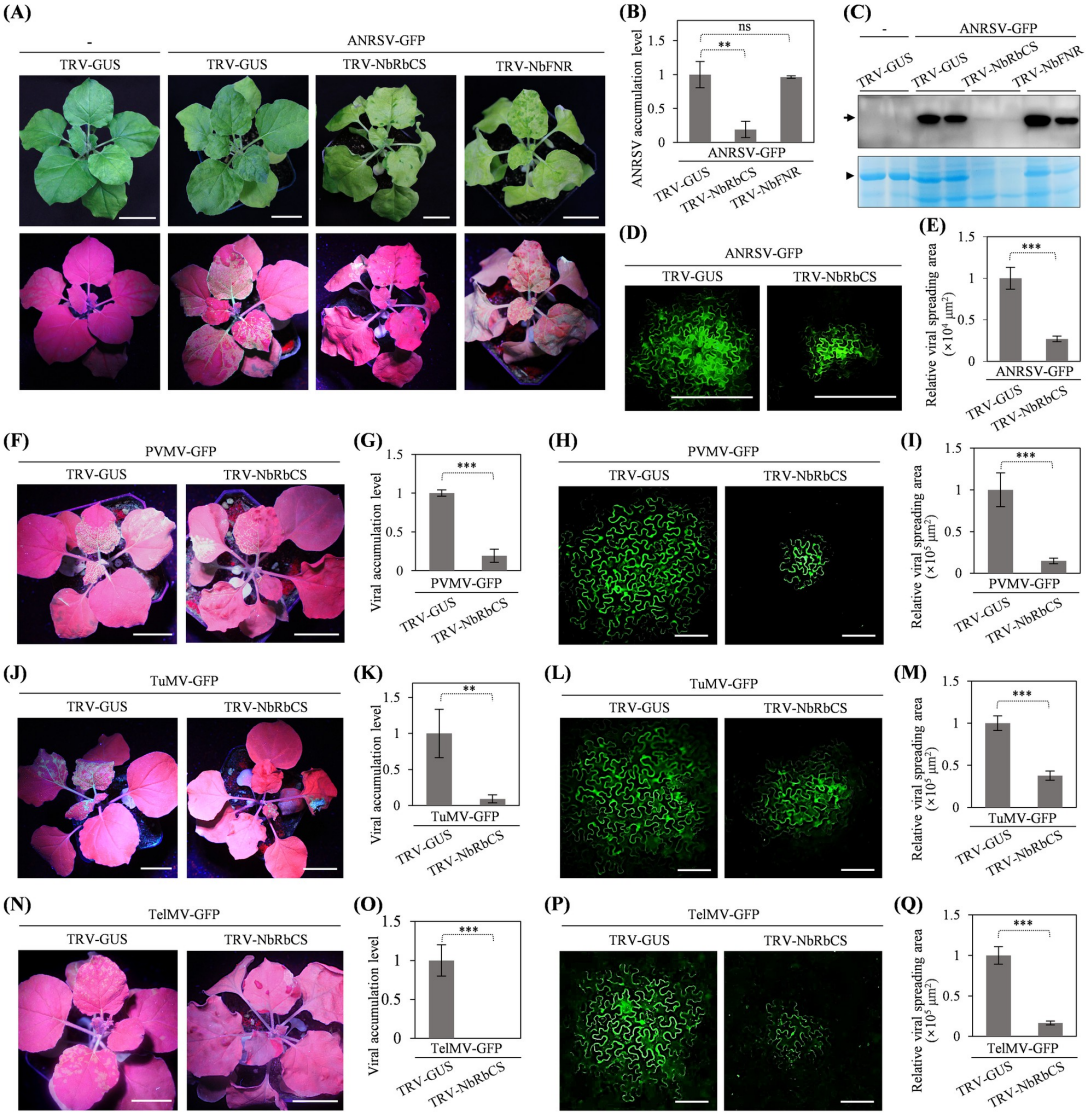
Knockdown of *NbRbCS* largely inhibits viral intercellular movement and systemic infection for ANRSV and other three tested potyviruses. (A) Silencing of *NbRbCS* significantly restricts ANRSV infection. *N*. *benthamiana* seedlings at 3- to 5-leaf stage were pre-inoculated with TRV-GUS, TRV-NbRbCS or TRV-NbFNR. At 12 dpi, these plants were challenged with ANRSV-GFP via sap rub-inoculation. The representative plants were photographed under daylight and UV light at ten days post-challenging inoculation (dpci). Bars, 2.5 cm. (B) Real-time RT-qPCR analysis of viral genomic RNA accumulation. Leaf samples were collected at 10 dpci for real-time RT-qPCR assay. Error bars denote the standard errors from three biological replicates. **, 0.001<*P*<0.01; ns, no significant difference. (C) Immunoblot analysis of GFP accumulation at 10 dpci. (D) Viral intercellular movement from single primarily infected cells at 108 hours post-challenging inoculation (hpci). Bars, 100 μm. (E) Statistical analysis of the size of viral infection foci at 108 hpci. For each treatment, a total of 25 infection foci from a total of six plants in three independent experiments were analyzed by ImageJ. The size of infection foci is presented as the mean ± SD (*n* = 25). The average value for TRV-GUS / ANRSV-GFP was designated 1×10^4^ μm^2^ to normalize the data. ***, *P*<0.001. (F, J, N) The effects of *RbCS*-silencing on the infectivity of three potyviruses. The representative plants were photographed under UV light at 6 dpci for PVMV-GFP (F) and TuMV-GFP (J), and at 13 dpci for TelMV-GFP (N). Bars, 2.5 cm. (G, K, O) Real-time RT-qPCR analysis of viral genomic RNA accumulation. Viral genomic RNA accumulation was determined at 6 dpci for PVMV-GFP (G) and TuMV-GFP (K), and at 13 dpci for TelMV-GFP (O). Error bars denote the standard errors from three biological replicates. **, 0.001<*P*<0.01; ***, *P*<0.001. (H, L, P) Viral intercellular movement from single primarily infected cells. Viral intercellular movement was recorded at 108 hpci for PVMV-GFP (H), and at 84 hpci for TuMV-GFP (L) and TelMV-GFP (P). Bars, 100 μm. (I, M, Q) Statistical analysis of the size of viral infection foci. The infection foci were determined at 108 hpci for PVMV-GFP (I) and at 84 hpci for TuMV-GFP (M) and TelMV-GFP (Q). For each treatment, a total of 20 infection foci from a total of six plants in three independent experiments were analyzed. The size of infection foci is presented as the mean ± SD (*n* = 20). The average value for control groups was designated 1×10^5^ μm^2^ to normalize the data. ***, *P*<0.001.

The effects of NbRbCS on ANRSV infection were investigated. *N*. *benthamiana* seedlings (*n* = 8 per clone) were pre-inoculated with TRV-NbRbCS, TRV-GUS or TRV-NbFNR (the parallel control). At 12 dpi, *NbRbCS* or *NbFNR* mRNA transcripts were significantly reduced (S10 Fig). Immediately, these plants were challenged with ANRSV-GFP via sap rub-inoculation. Ten days later, strong fluorescence signals, indicative of ANRSV-GFP infection, were observed in upper leaves of all pre-treated plants with TRV-GUS or TRV-NbFNR, whereas *NbRbCS*-silenced plants exhibited scattered fluorescence signals along veins (Fig 9A). Real-time RT-qPCR and immunoblotting assays confirmed that ANRSV infection was largely restricted in *NbRbCS*-silenced plants (Fig 9B and 9C). The effects of *NbRbCS* on viral intercellular movement were also examined. Agrobacterial culture harboring pRS-G (OD_600_ = 0.001) was inoculated into *NbRbCS*-silenced and control plants. At 108 hpi, the size of viral spreading from primarily-transfected to peripheral cells was much smaller in *NbRbCS*-silenced leaves (Fig 9D and 9E). We employed a similar strategy to test the effects of NbRbCS on viral infectivity for other three potyviruses, pepper veinal mottle virus (PVMV), telosma mosaic virus (TelMV), and TuMV. The results showed that *NbRbCS*-silencing significantly impairs systemic infection and intercellular movement for PVMV (Fig 9F–9I), TuMV (Fig 9J–9M) and TelMV (Fig 9N–9Q), indicating that NbRbCS plays a general regulatory role in potyvirid infection.

## Discussion

**Fig 10:**
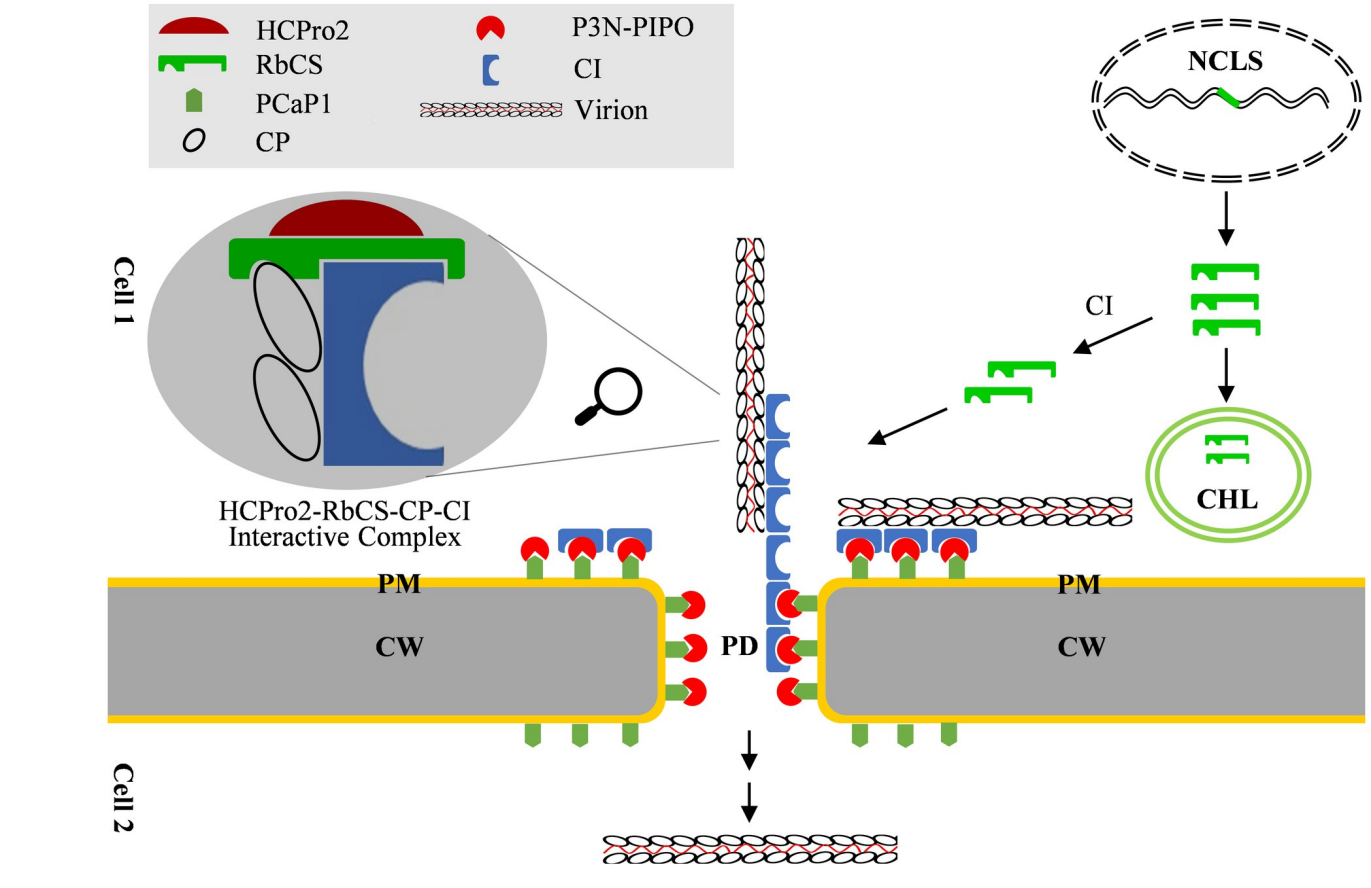
A working model depicting that NbRbCS is co-opted as the scaffold protein in mediating the assembly of viral intercellular movement complex. NCLS, nucleus; CHL, chloroplast; PM, plasma-membrane; CW, cell wall; PD, plasmodesmata.

HCPro assists viral movement, but how does it connect with intercellular movement has been not demonstrated. This study provides genetic and biochemical evidence supporting a role of HCPro2 (a homolog of potyviral HCPro) in viral intercellular movement. HCPro2, together with CI and CP, form the complexes at PD. The interactions among them do not involve cell membranes, but are indeed facilitated by a host protein NbRbCS, abundant in a plant cell. The fraction of RbCS involved in the interactions is distinct from the chloroplast pool. Knockdown of *NbRbCS* by gene silencing impairs their interactions, and viral intercellular movement and systemic infection. Therefore, we envisage a scenario that the nucleus-encoded RbCS is hijacked as a pro-viral factor to mediate the assembly of intercellular movement complex to promote viral cell-to-cell movement (Fig 10). The model might be generally applied to other potyvirids, based on following considerations: i) The interactions among the three viral factors (HCPro, CI, and CP) have been documented for numerous potyvirids, however, these interactions were usually detected *in planta*, rarely *in vitro* [28–31,69–77], suggesting a potential role of RbCS in mediating these interactions; ii) Silencing of *NbRbCS* significantly attenuates the cell-to-cell movement for ANRSV but also other three tested potyviruses.

The genomic 5′-terminal regions of potyvirids encode two types of leader proteases: serine-protease (P1) and cysteine-protease (HCPro), which differ greatly in the arrangement and sequence composition among inter-genus viruses [18,78]. One of leader proteases expresses RSS activity for each potyvirid. The arepaviruses have two copies of HCPro (HCPro1-HCPro2), with HCPro2 as the VSR. HCPro1 is dispensable for ANRSV infection in *N*. *benthamiana*. The lethality of HCPro1 deletion in ANSSV [63] might be explained by the fact that *N*. *benthamiana* is less susceptible to ANSSV [79]. The phenomenon that the leader protease without RSS activity is dispensable has been reported for several potyvirids [80–82]. Although the HCPro of wheat streak mosaic virus with loss-of-RSS activity is dispensable for viral infection, it is a determinant in eriophyid mite-vectored transmission [83]. In the case of plum pox virus (PPV), P1 protein is not essential during viral infection, but it elaborately modulates viral replication to evade host immune response [81]. Here, we attempt to speculate that HCPro1 might play an accessory role in viral infection or function in insect-vectored transmission.

This study performed a comprehensive investigation on ANRSV HCPro2, and provided substantial evidence to support its role in cell-to-cell movement: i) Replacement of HCPro2 with an unrelated VSR (P19) does not affect viral RNA accumulation, but nearly abolishes viral cell-to-cell movement. ii) Substitution of HCPro2 with its counterpart from a potyvirus efficiently complements viral intercellular movement, indicating that inter-genus HCPros might be functionally interchangeable in aiding viral intercellular movement. iii) Movement-related proteins CI and CP are co-purified with HCPro2. iv) HCPro2, CI and CP form the PD-targeting complexes, which is pivotal in viral cell-to-cell movement. The above results, together with previous observations [37] and the fact that HCPro interacts with CI or/and CP *in planta* for numerous potyvirids [26,36,84,85], suggest that different potyvirid HCPros might share a common function in aiding viral intercellular movement. Nevertheless, the underlying molecular mechanism is still unknown at this time. Previous studies revealed that both PPV and PVA HCPros have the capacity to stabilize CP and enhance the yield of viral particles [38,39], suggesting that HCPro aids viral intercellular movement in an indirect manner. Intriguingly, the steady-state of CP mediated by HCPro was observed either in a context of viral infection or in the presence of viral proteins P3-to-CP, whereas the co-expression of HCPro and CP does not [38,39]. Consequently, we propose that HCPro facilitates to stabilize CP and enhance viral particle yield likely via the formation of HCPro-RbCS-CP-CI complex (Fig 10).

HCPro2 is distributed, with a varied degree, into different cellular compartments in viral infection. The HCPro2-formed inclusions mainly targeted to PD, but a small portion of them are elsewhere. In recent years, a significant progress has been achieved with regard to the aggregates induced by PVA HCPro. The aggregates (called as PVA-induced granules, PGs) are multifunctional during viral infection, including viral genome translation, RSS, encapsidation and systemic spread [86–88]. Whether the HCPro2 inclusions that are not targeted to PD behave similar functions to PGs awaits to be investigated. Among five viral proteins co-purified with HCPro2, four (P3, 6K1, CI, and NIb) are components of 6K2-induced replication complex [19,84]. HCPro was also identified from 6K2-induced replication vesicles for PVA [89]. It is logical to speculate that HCPro2 might also participate in viral replication, which would be a promising research direction.

The chloroplast has long been recognized as a common target by many plant viruses. Plant viruses may directly modify chloroplast membranes to assemble viral replication complex, or co-opt chloroplast proteins for viral replication, movement or/and counteracting host defense. The rubisco is highly expressed in plants, and believed to be the most abundant protein on the planet [90]. However, only one document is dedicated to the description of RbCS-virus interaction and its biological relevance [57]. In this study, we provide multi-disciplinary evidences to support that RbCS is co-opted to mediate multiple interactions among viral movement-related proteins, likely functioning in the assembly of movement complex (Fig 10). Here, we discuss five critical points that need to be clarified in future: i) How is RbCS recruited to PD? Potyvirid CI, when expressed alone *in planta*, is localized in cytoplasm in the form of irregular aggregates. Once P3N-PIPO is co-expressed, CI is recruited to PD and forms cone-shaped structures [20]. Thus, it is speculated that CI might recruit RbCS to PD via the interaction in viral infection (Fig 10). ii) Whether the multiple interactions among HCPro2, RbCS, CP, and CI (including potential self-interactions) have synergistic enhancement effect awaits to be investigated. iii) It is so fascinating that RbCS, such a small molecule, interacts with three viral movement-related proteins. In the RbCS-mediated complex, it is unclear whether one molecule of RbCS simultaneously interacts with HCPro2, CI and CP, or more molecule are needed. To clarify this point, a fine mapping of interaction sites between RbCS and HCPro2, CI or CP should be performed. iv) Why would a variety of viral factors and host proteins be needed for the intercellular movement of potyvirids? As stated in introduction, three viral factors (CI, CP, and P3N-PIPO), together with HCPro2 or HCPro identified in this study, participate in viral intercellular movement, although the actual roles of them have not been clearly defined. Noticeably, these proteins do not contain a typical transmembrane domain. How could they be translocated to PD to facilitate viral intercellular movement? In line with this point, several plasma membrane- or PD-localized proteins were identified to be potentially involved in this event [10,43–45]. As depicted in the model (Fig 10), it is very possible that P3N-PIPO is anchored to PD by a cellular membranous protein—PCaP1 [10]. The CI is recruited to PD by P3N-PIPO [20]. RbCS is co-opted to act as a mediator to aggregate both HCPro2 and CP/virion at PD. v) It is worth noting that RbCS interacts with tobamoviral MPs to facilitate viral intercellular and long-distance movement, although the underpinning molecular mechanism was not reported [57]. It seems that plant viruses likely evolved different strategies to utilize such an abundant chloroplast protein in viral infection, which deserves more studies in the future.

A previous report showed that RbCS interacts with P3 for several potyviruses [58]. Besides P3, other three protein (HCPro1, VPg and NIb) of ANRSV interact with RbCS (S9 Fig). Coincidentally, two of them (P3 and NIb) are co-purified with HCPro2. VPg plays multifunctional roles during viral infection. Among them, VPg is targeted to membranous factories and plays a key role in viral replication [91,92]. Taken together, we envisage that RbCS might also participate in viral replication via its interactions with replication-related viral proteins. Again, it is amazing that an abundant chloroplast protein has the capacity of interaction with multiple viral proteins. A fine mapping of interaction sites among them might help design resistance strategy of conferring broad resistance to potyvirids.

## Materials and methods

### Plant materials and virus resources

*N*. *benthamiana* plants were maintained in a growth cabinet set under the conditions of 16 h of light at 25°C and 8 h of darkness at 23°C, with 70% relative humidity. In sap rub-inoculation assays, homogenates containing GFP-tagged telosma mosaic virus (TelMV-GFP), pepper veinal mottle virus (PVMV-GFP) and TuMV-GFP were prepared from infected leaf tissues of *N*. *benthamiana* plants pre-inoculated with pPasFru-G, pHNu-GFP and pCBTuMV-GFP/mCherry, respectively [35,93,94]. An infectious cDNA clone of ANRSV-ZYZ (pRS), as well as its derivative pRS-G (GFP-tagged ANRSV clone) were previously developed [79].

### Development of ANRSV-derived cDNA clones

Either pRS-G or pRS-G was used as the backbone to construct a series of ANRSV-derived virus clones, including pRS-G(ΔHCPro1), pRS-G(ΔHCPro2), pRS-G(tuHCPro), pRS-G(tbP19), pRS-GFP-HCPro2, pRS-G-2×Strep-HCPro2 and pRS-G(ΔHCPro1)-2×Strep-HCPro2. These clones were constructed by a similar strategy, mainly based on standard DNA manipulation technologies such as overlapping PCR. Herein, the detailed description for the creation of pRS-G(ΔHCPro2), in which the complete HCPro2-coding sequence in ANRSV was deleted, was stated. Two PCR reactions with pRS-G as the template were performed using corresponding primer sets PCB301-F/SOE-HP2-R and SOE-HP2-F/RSV-3-R (S2 Table). A mixture of resulting PCR products was used as the template for overlapping PCR with primer set PCB301-F/ RSV-3-R (S2 Table). The obtained fragment was inserted back into pRS-G by using *Pme* I / *Mlu* I sites to generate pRS-G(ΔHCPro2). The pRS-G(ΔGDD), a replication-defective virus clone, was created via the removal of strictly-conserved GDD motif in viral RNA polymerase (NIb). Two fragments upstream and downstream of GDD motif in pRS-G were amplified with corresponding primer sets RSV-5-F/SOE-GDD-R and SOE-GDD-F/RSV-5-R (S2 Table), and then mixed as the template for overlapping PCR with primer set RSV-5-F/RSV-5-R (S2 Table). The obtained fragment was inserted back into *EcoR* I / *Sal* I-treated pRS-G to generate pRS-G(ΔGDD).

### Plasmids construction

For the RSS assay, four plasmids, including pCaM-HCPro1-HCPro2-HA, pCaM-HCPro1-HA, pCaM-HCPro2-HA, and pCaM-ssHCPro2-HA, were constructed for respective expression of HCPro1-HCPro2-HA, HCPro1-HA, HCPro2-HA and ssHCPro2-HA. The coding regions of them were amplified from pRS-G or pSS-I-G, and individually integrated into a binary plant expression vector pCaMterX [95] by using *Xho* I / *Kpn* I sites. The complete sequences of *NbRbCS* and *NbFNR* sequences are deposited in NCBI GenBank database with accession numbers as QCS40508.1 and QAV53876.1. We referred to these sequences to design primers in this study (S2 Table). For TRV-based VIGS analysis, SGN VIGS Tool (https://vigs.solgenomics.net) was employed to design two pairs of primers TRV-NbRbCS-F/TRV-NbRbCS-R and TRV-NbFNR-F/TRV-NbFNR-R (S2 Table) for amplifying two ~300 bp-fragments corresponding to *NbRbCS* and *NbFNR*. The obtained fragments were individually cloned into pTRV2 [96] by utility of *BamH* I / *Xho* I sites to obtain pTRV2-NbRbCS and pTRV2-NbFNR (S2 Table). For Y2H, BiFC and Co-IP assays, the corresponding plasmids were generated by using Gateway cloning technology. Briefly, the coding sequences of indicated cistrons were engineered into the entry clone—pDONR221, and then transferred into the desired gateway-compatible destination vectors, including pGADT7-DEST, pGBKT7-DEST, pEarleygate201-YN, pEarleygate202-YC, and pBA-FLAG-4myc-DC [97–99]. In addition, we constructed four plasmids (pCaM-GFP-HCPro2, pCaM-GFP-CP, pCaM-CI-mCherry, pCaM-CP-mCherry, and pCaM-NbRbCS-mCherry) for respective expression of GFP-HCPro2, GFP-CP, CI-mCherry, CP-mCherry, and NbRbCS-mCherry. For them, we amplified complete *GFP* and *mCherry* sequences from pVPH-GFP//mCherry [100], individually engineered them to pCaMterX, and obtained two intermediate vectors—pCaM-GFP and pCaM-mCherry. Then, the coding sequences of HCPro2, CI, CP, and NbRbCS were individually integrated into pCaM-GFP or pCaM-mCherry to produce the four plasmids via seamless cloning or restriction endonuclease digestion-T4 DNA ligation strategy. For MYTH assay, the HCPro2-coding sequence was integrated into the bait vector—pBT3-STE by using *Sfi* I site to produce pBT3-STE-HCPro2 for the expression of HCPro2-Cub-LexA. CI, CP and P3N-PIPO were individually cloned into the prey vector—pPR3-N(DEST) [101] via Gateway cloning technology for respective expression of Nub-CI, Nub-CI, and Nub-P3N-PIPO.

All plasmids in this study were verified by Sanger DNA sequencing.

### Agroinfiltration and sap rub-inoculation

*Agrobacterium* (strain GV3101)-mediated transformation was performed following previous description [63,79]. Fully expanded leaves of *N*. *benthamiana* seedlings were infiltrated with agrobacterial cultures harboring relevant plasmids. *N*. *benthamiana* seedlings at 3- to 5-leaf stage were used for infectivity test of ANRSV-derived cDNA clones. The seedlings at 6- to 8-leaf stage were used for transient expression of genes of interest. For TRV-VIGS assays [96], two agrobacterial cultures harboring pTRV1 along with pTRV2-GUS (TRV-GUS), pTRV2-NbRbCS or pTRV2-NbFNR were mixed (final OD_600_ = 0.3 per culture), and infiltrated into *N*. *benthamiana* seedlings at 3- to 5-leaf stage. Sap rub-inoculation assays were essentially performed according to a previously described protocol [79].

### Y2H and MYTH

Yeast two-hybrid (Y2H) assays were performed according to the Yeastmaker Yeast Transformation System 2 User Manual (Clontech). Each pair of indicated genes were cloned into pGBKT7-DEST for fusing with GAL4 DNA binding domain (BD) or pGADT7-DEST for fusing with GAL4 activation domain (AD). Yeast competent cells (Y2H Gold) was co-transformed with bait and prey constructs, followed by 10-fold serial dilution and plating onto synthetic defined (SD) yeast leucine and tryptophan dropout medium (SD/-Leu/-Trp) or leucine, tryptophan, histidine and adenine dropout medium (SD/-Leu/-Trp/-His/-Ade). The transformants were allowed by 4- to 6-day growth on the dropout mediums at 28°C. For immunoblot detection of protein expression in yeast, the co-transformed yeast cells were propagated in liquid SD/-Leu/-Trp medium, and the cultures were harvested at OD_600_ of 0.5. The total proteins were extracted by using Yeast Protein Extraction Reagent (Takara), followed by immunoblot detection with anti-HA monoclonal or anti-Myc polyclonal antibody (Abcam). Membrane yeast two hybrid (MYTH) assays were exactly performed according to the user manual of DUALmembrane starter kits (Dualsystems Biotech). Yeast competent cells (NMY51) were co-transformed with each pair of the indicated constructs, and plated onto SD/-Leu/-Trp and SD/-Leu/-Trp/-His/-Ade mediums.

### BiFC

Each pair of indicated genes were integrated into pEarleyGate201-YN and pEarleyGate202-YC [98] for the expression of desired proteins fused with N-terminal half (YN) or C-terminal half of YFP (YC). Two agrobacterial cultures harboring YN- or YC-constructs were mixed (final OD_600_ = 0.3 per culture), and infiltrated into fully developed leaves of *N*. *benthamiana*. The inoculated leaves were examined by an inverted fluorescence microscope (BX53, OLYMPUS) at the indicated time points. To relatively quantify the intensity of protein-protein interaction among different treatments by BiFC, the YFP fluorescence signals were captured when all the conditions, i.e., 10×objective, U-FBNA filter (BP470-495; BA510-550), burner status set: 50% or 100% (U-HGLGPS), and the value of exposure time, were kept constant.

### Co-IP

Total proteins were extracted from one gram of co-inoculated leaves of *N*. *benthamiana* by using 2 mL of ice-cold immunoprecipitation buffer (10% [v/v] glycerol, 25 mM Tris-HCI, pH 7.5, 150 mM NaCl, 10 mM DTT, 1 mM EDTA, 1 × Protease Inhibitor Cocktail, For Plant Cell (Sangon Biotech), and 0.15% [v/v] Nonidet P-40). Protein extracts were incubated with GFP-Trap beads (ChromoTek) for 1h at 4°C. The beads were collected and washed with the buffer (10 mM Tris-HCl pH 7.5, 150 mM NaCl, 0.05% Nonidet P40, 0.5 mM EDTA). Total protein extracts prior to (Input) and after immunoprecipitation (IP) were analyzed by immunoblotting using anti-GFP and anti-Myc polyclonal antibodies (Abcam), essentially as previously described [63].

### Streptavidin affinity purification and LC-MS/MS

The upper non-inoculated leaves were collected from *N*. *benthamiana* plants infiltrated with pRS-G-2×Strep-HCPro2, pRS-G(ΔHCPro1)-2×Strep-HCPro2 or pRS-G at 12 dpi. Streptavidin affinity purification, SDS-PAGE and immunoblot analysis, and LC-MS/MS identification were conducted essentially as described by Hu and colleagues [102].

### Subcellular fractionation assay

Several previous documents were referred to perform subcellular fractionation assay [103–105]. One gram of leaf tissues per treatment were fine homogenized in 4 mL of lysis buffer (50 mM Tris-HCl, pH 7.4, 15 mM MgCl_2_, 10 mM KCl, 20% glycerol, 1 × Protease Inhibitor Cocktail). The homogenate was centrifuged at 1000 *g* for 5 min at 4°C to remove the debris, and the supernatant (S1) was obtained. S1 was centrifuged at 3700 *g* for 10 min at 4°C, resulting in supernatant (S3) and crude pellet (P3). P3 fraction includes nuclei, chloroplasts and cell wall. S3 was centrifuged at 30000 *g* for 50 min at 4°C to separate soluble (S30) and crude membrane (P30). Both P3 and P30 pellets were resuspended in the lysis buffer (4 mL per pellet). An aliquot of 10 μL per sample was used for immunoblot analysis.

### Northern blot and real-time RT-qPCR

Norther blot assays were performed to detect *GFP* mRNA abundance, essentially as previously described by Qin and colleagues [63]. Real-time RT-qPCR was employed to relatively quantity viral genomic RNAs or endogenous gene transcripts, following a previously described protocol by Hu and colleagues [102]. The primers used in the assays were listed in S2 Table.

### Aniline blue staining

Aniline blue solution is prepared before use via mixing 0.1% aniline blue (Sigma-Aldrich)-water solution with 1 M glycerol solution in a ratio of 2:3. The mixture was infiltrated into *N*. *benthamiana* leaves by using a 1 mL needle-free syringe. Thirty minutes later, aniline blue fluorescence was observed under confocal microscope.

### Confocal microscopy

The epidermal cells of inoculated leaves with relevant plasmids were observed under a confocal microscopy (FV1000, OLYMPUS) with a 20× water immersion objective. Excitation wavelengths and emission filters were 488 nm/bandpass 500–530 nm for GFP or YFP, 543 nm/bandpass 580–620 nm for mCherry, and 405 nm/band-pass 442–472 nm for aniline blue fluorochrome.

## Supporting information

S1 Data Excel spreadsheet containing, in separate sheets, the underlying numerical data and statistical analysis for Figure panels 1D, 2D, 2E, 7E, 7H, 7K, 9B, 9E, 9G, 9I, 9K, 9M, 9O, 9Q, S3C, S4, S10B and S10C. (XLSX)

S1 Table List of host proteins that are uniquely identified from co-purified products with 2×Strep-HCPro2 by LC-MS/MS. (PDF)

S2 Table List of primers used in this study. (PDF)

S1 Fig RT-PCR detection of ANRSV and its derivatives. The upper non-inoculated leaves of *N*. *benthamiana* plants were assayed at 16 dpi. RT-PCR was conducted with primer set 8900F/9300R (S2 Table) that target viral *CP* region.
(TIF)

S2 Fig RNA silencing suppression test of HCPro1, HCPro2, and HCPro1-HCPro2 of ANRSV. (A) Representative photographs of co-infiltrated *N*. *benthamiana* leaves were taken under UV light at 72 hpi. Each of three plasmids (for the transient expression of HCPro1-HA, HCPro2-HA, HCPro1-HCPro2-HA, respectively), together with a GFP-expressing plasmid, were co-inoculated into *N*. *benthamiana* leaves via agroinfiltration. Co-expression of GFP along with either empty vector—pCaMterX (Vec) or HA-tagged ANSSV-encoded HCPro2 (ssHCPro2-HA) were included as negative and positive controls, respectively. (B) Immunoblot detection of GFP accumulation in co-inoculated leaf patches at 72 hpi. Coomassie blue staining of RbCL was used as a loading control. (C) Northern blot analysis of *GFP* transcript accumulation in co-inoculated leaf patches at 72 hpi. Ethidium bromide staining of ribosomal RNA (rRNA) was served as a loading control.
(TIF)

S3 Fig The effects of deletion of HCPro2 or its substitution with different VSRs on viral infectivity. (A) Infectivity test of pRS-G and its derivatives in *N*. *benthamiana*. Representative photographs were taken under UV light at 13 dpi and 30 dpi. The leaf region indicated by dashed box is enlarged. Mock, empty vector control. Bars, 5 cm. (B) The observation of viral cell-to-cell movement for the indicated virus clones at 60 hpi and 84 hpi. Bars, 100 μm. (C) Statistical analysis of the size of viral spreading area at 84 hpi. For each clone, at least 25 infection foci from a total of six plants in three independent experiments was analyzed. The size of infection foci is calculated by ImageJ. The data are presented as the mean ± SD (*n* ≥ 25). The average value for wild-type pRS-G was designated 1×10^5^ μm to normalize the data. **, 0.001<*P*<0.01.
(TIF)

S4 Fig Real-time RT-qPCR analysis of viral genomic RNA accumulation. The upper non-inoculated leaves of *N*. *benthamiana* plants were sampled at 12 dpi for the assay. RT-qPCR with primer set RS9200F/RS9350R (S2 Table) targeting viral *CP* region was performed. Error bars denote the SD from three biological replicates. The average value for pRS-G-2×Strep-HCPro2 was designated 1.0 to normalize the data. *, 0.01<*P*<0.05; **, 0.001<*P*<0.01.
(TIF)

S5 Fig Immunoblot analysis of the expression of BD- and AD-fused proteins in yeast. The bands, indicated by red asterisks, correspond to the predicted size of recombinant proteins (~54.5 kDa for AD-HCPro2, 97.87 for BD-CI, 57.46 for BD-P3N-PIPO, and 54.51 for BD-CP). The arrowhead, AD-HCPro2. Coomassie blue staining of the total proteins (CBB) was used as a loading control.
(TIF)

S6 Fig Y2H tests the interactions of HCPro2 with six viral proteins. Yeast competent cells (Y2H Gold) were co-transformed to express the indicated pairs of proteins. The transformed cells were subjected to 10-fold serial dilutions and plated on SD/-Trp/-Leu and SD/-Trp/-Leu/-His/-Ade mediums. The plates were cultured at 28°C for four to six days before photographing.
(TIF)

S7 Fig MYTH tests the interactions of HCPro2 with CI, CP and P3N-PIPO. Yeast competent cells (NMY51) were co-transformed to express the indicated pairs of proteins. The transformed cells were subjected to 10-fold serial dilutions and plated on SD/-Trp/-Leu and SD/-Trp/-Leu/-His/-Ade mediums. The plates were cultured at 28°C for four to six days before photographing. Co-transformation of a pair of constructs for simultaneous expression of soybean mosaic virus (SMV) P3-Cub-LexA and Nub-EIF4A [101] was included as the positive control.
(TIF)

S8 Fig NbRbCS interacts with both N2 and D2 domains of HCPro2. (A) Schematic diagram of HCPro2 showing N2 and D2 domains. The red box represents the cysteine protease domain of HCPro2. (B) The interactions of NbRbCS with N2 and D2 domains were tested by Y2H assays. The coding sequence of NbRbCS was cloned into pGADT7-DEST and pGBKT7-DEST for respective expression of GAL4 AD-fused (AD-NbRbCS) and BD-fused NbRbCS (BD-NbRbCS). The coding sequences of N2 and D2 domains of HCPro2 were cloned into pGADT7-DEST for respective expression of AD-fused N2 (AD-N2) and D2 (AD-D2), and cloned into pGBKT7-DEST for respective expression of BD-fused N2 (BD-N2) and D2 (BD-D2). The co-transformed yeast cells for co-expressing the indicated pairs of proteins / domains were subjected to 10-fold serial dilutions and plated on SD/-Trp/-Leu/-His/-Ade mediums. (C) The interactions of NbRbCS with N2 and D2 domains were tested by BiFC assays. The coding sequences of N2 and D2 were individually integrated into pEarleyGate201-YN for expressing YFP YN-fused N2 (N2-YN) and D2 (D2-YN). *N*. *benthamiana* leaves were co-inoculated for the expression of indicated pairs of proteins. YFP signals (shown in green) were observed by fluorescence microscope at 72 hpi. The co-expression of YC and the indicated protein was included as the negative controls. Bars, 100 μm.
(TIF)

S9 Fig Y2H tests the interaction of NbRbCS with seven viral factors. The co-transformed yeast cells for co-expressing the indicated pairs of proteins were subjected to 10-fold serial dilutions and plated on SD/-Trp/-Leu and SD/-Trp/-Leu/-His/-Ade mediums. Co-transformation of yeast cells for simultaneous expression of AD-T7-T and BD-T7-53 was included as the positive control.
(TIF)

S10 Fig Silencing of *NbRbCS* or *NbFNR* in *N*. *benthamiana*. (A) Phenotypic observation of *NbRbCS*- or *NbFNR*-silenced in *N*. *benthamiana*. *N*. *benthamiana* seedlings at 3- to 5-leaf stage were inoculated with pTRV1 along with pTRV2-NbRbCS (TRV-NbRbCS) or pTRV2-NbFNR (TRV-NbFNR), and photographed at 12 dpi. Co-inoculation of pTRV1 and pTRV2-GUS was included as the parallel control. Bars, 2.5 cm. (B, C) Real-time RT-qPCR analysis of *NbRbCS* or *NbFNR* mRNA transcript accumulation. The samples were collected at 12 dpi for the assay. Error bars denote the standard errors from three biological replicates. The average value for TRV-GUS was designated 1.0 to normalize the data. ***, *P*<0.001.
(TIF)

S11 Fig Immunoblot analysis of co-expression of the indicated proteins in *NbRbCS*- and *NbFNR*-silenced plants. The co-inoculated leaves for co-expression of HCPro2-YN / CI-YC (A), HCPro2-YN / CP-YC (B) or CI-YN / CP-YC (C) were sampled at 60 hpi (B) or 72 hpi (A, C) for immunoblot analysis using anti-GFP antibody. As the abundance of RbCL was greatly decreased along with *RbCS*-silencing, Coomassie blue staining of protein bands (indicated by red asterisks) was used as a loading control.
(TIF)

S12 Fig NbRbCS(ΔCTP) interacts with HCPro2, CI and CP. (A) Schematic diagram of NbRbCS(ΔCTP). NbRbCS(ΔCTP) is a truncated version of NbRbCS, with a removal of chloroplast transit peptide (CTP). (B) Y2H tests the interactions of NbRbCS(ΔCTP) with HCPro2, CI and CP. The transformed yeast cells for co-expression of the indicated proteins were subjected to 10-fold serial dilutions and plated on SD/-Trp/-Leu/-His/-Ade mediums. Co-transformation of a pair of constructs for the expression of AD-T7-T and BD-T7-53 was included as the positive control. (B) BiFC assay tests the interactions of NbRbCS(ΔCTP) with HCPro2, CI and CP. *N*. *benthamiana* leaves were co-inoculated for the expression of the indicated combination of proteins. YFP signals (shown in green) were observed by fluorescence microscope at 72 hpi. Bars, 50 μm.
(TIF)
