# Supplementary material for: Rubisco small subunit (RbCS) is co-opted by potyvirids as the scaffold protein in assembling a complex for viral intercellular movement
Source: PLoS Pathog. 2024 Mar 4;20(3):e1012064. doi: 10.1371/journal.ppat.1012064 (PMC10939294; doi:10.1371/journal.ppat.1012064)
Supplement: S1 Table — (PDF) [file ppat.1012064.s002.pdf]

S1 Table. List of host proteins that are uniquely identified from co-purified products with 2×Strep-HCPro2 by LC-MS/MS

No.	Uniprot	GenBank	Protein description	Score
1	A0A6H0CCF4	QIS68115.1	ATP synthase subunit alpha	198.61
2	Q2LAH1	ABC59515.1	Chloroplast photosystem II 22 kDa component	96.91
3	H9A0F9	AFC90091.1	Eukaryotic initiation factor 4A-14	92.86
4	I3QHX5	AFK08605.1	Adenosylhomocysteinase	74.35
5	A0A0F7R532	BAR72289.1	S-adenosylmethionine synthase 1a	58.38
6	E5LLE7	ADR71054.1	Phosphoglycerate kinase	51.82
7	Q6XX19	AAP04393.1	Translation elongation factor 1 alpha (Fragment)	50.78
8	B8R6B6	ACJ61779.1	ELI3 (Fragment)	50.39
9	A0A0A8K9V3	CAE47826.1	Geranylgeranyl reductase	47.88
10	Q5EC25	AXN93983.1	Ubiquitin	47.67
11	A0A0S4IJL0	QCS40508.1	Ribulose bisphosphate carboxylase small subunit, chloroplastic	29.14
12	A0A088F9I1	AIM41268.1	Chloroplast ATP-dependent Clp protease chaperone protein	27.61
13	R9W4N2	AGN92480.1	Thioredoxin-dependent peroxiredoxin (Fragment)	26.43
14	A0A387K371	BBG12274.1	GTP-binding nuclear protein	25.25
15	U3MY90	AGW21713.1	Proteinase inhibitor (Fragment)	24.80
16	Q6L9F6	BAD22854.1	Heat shock protein 70 (Fragment)	24.62
17	K0IBB4	AFU48609.1	Catalase (Fragment)	23.93
18	Q5EFR5	AAW80966.1	Chloroplast oxygen-evolving protein 16 kDa subunit, psbQ	23.12
19	I0B7J1	AAX53163.1	Chloroplast photosynthetic oxygen-evolving protein 33 kDa subunit, psbO	22.13
20	Q5XPZ0	AAU93700.1	Adenosine kinase (Fragment)	20.11
21	A0A387K491	BBG12268.1	Ran binding protein RanBP1-1b	17.76
22	A0A0F7JLU6	AKH15660.1	Glyceraldehyde-3-phosphate dehydrogenase 1	17.48
23	E0X585	ADM18296.1	Glycine dehydrogenase (aminomethyl-transferring)	16.94
24	A0A0S0N5Y9	ALH22047.1	Tubulin alpha chain 6	16.89
25	E1AXT8	ADM26718.1	(S)-2-hydroxy-acid oxidase	16.75
26	Q2QFR3	AAZ32410.1	Cysteine proteinase aleuran type	16.37
27	A0A173FEI6	ANG84008.1	ATP-dependent RNA helicase eIF4a	15.65
28	Q5EEQ1	AAW83128.1	Photosystem I reaction center subunit X	14.91
29	A0A248QEL2	ASP44115.1	S-adenosylmethionine synthase	14.36
30	A0A0H5BGR5	BAR94039.1	Acetoacetyl-coenzyme A thiolase 1	14.14
31	Q52JJ5	AAY18610.1	Glutamyl-tRNA synthetase	14.08
32	J7EYL4	AER93282.1	GRX1	13.08
33	D5LT98	ADF43794.1	Chloroplast elongation factor TuB (Fragment)	12.17
34	Q6RI18	AAS75819.1	Isopentenyl/dimethylallyl diphosphate isomerase (Fragment)	12.08
35	C9DFA3	ACV52574.1	FtsH-like protein (Fragment)	12.00
36	K7ZLE1	BAM66423.1	Calcium-sensing receptor	11.75
37	M4T8W2	AGH70220.1	Cytosine-specific methyltransferase (Fragment)	11.08
38	A0ZVU2	CAF04055.2	S-adenosylmethionine transporter	10.29
39	A0A238ML06	SMZ59130.1	H(+)-exporting diphosphatase, NbPPA1.2	10.13
40	A0A7M1I5W8	QOQ34361.1	SBT8	10.08
41	A4D0J8	AAY17069.2	Carbonic anhydrase	9.805
42	V5KY72	AHA42253.1	Ubiquitin-conjugating enzyme variant	9.08
43	H9C956	AFD62806.1	Phospho-2-dehydro-3-deoxyheptonate aldolase (Fragment)	8.70
44	I1Y996	AFI98561.1	RabE1	8.69
45	Q2LAH0	ABC59516.1	Chloroplast photosystem II 22 kDa component, psbS1	8.59
46	A7IYM9	ABF74733.1	ADP-ribosylation factor 1	8.57
47	A0A387K3P5	BBG12272.1	GTP-binding nuclear protein, NbRan1a	8.20
48	Q7XZC7	CAE00191.1	Magnesium chelatase subunit (Fragment)	8.08
49	C9DFB6	ACV52587.1	FAD-binding FR-type domain-containing protein (Fragment)	7.88
50	A0A0A7HDA5	AIZ01224.2	Epi-aristolochene dihydroxylase	7.08
51	Q1WL50	ABA42892.1	Trypsin proteinase inhibitor	6.11
52	B2D077	ACB54686.1	Methyltransferase	6.08

Note: The candidate proteins with the score above 6.0 (calculated by MaxQuant) are regards as potential targets.
