# Supplementary material for: Rubisco small subunit (RbCS) is co-opted by potyvirids as the scaffold protein in assembling a complex for viral intercellular movement
Source: PLoS Pathog. 2024 Mar 4;20(3):e1012064. doi: 10.1371/journal.ppat.1012064 (PMC10939294; doi:10.1371/journal.ppat.1012064)
Supplement: S2 Table — (PDF) [file ppat.1012064.s003.pdf]

S2 Table. List of primers used in this study

Primer name	Sequence (5'→3')	Use
PCB301-F*	TACCCGCCAATATATCCTGTC	common primers for plasmid construction
RSV-3-R*	ATATA***GGATCC***TCCATGCATCCTGTCGCT	
RSV-5-F*	ATATA***GAATTC***GTTTACACACCAATTTACTGTGTGGAT	
RSV-5-R*	ATATA***GTCGAC***TTTTTTTTTTTTTTTTTTTTACCTAAGGGTAGTAGGTTCATTGA	
SOE-HP1-F	TTGCAGGGGCAACAATGGCACACCAAGATGAGGAAATG	pRS-G(ΔHCPro1)
SOE-HP1-R	CATCTTGGTGTGCCATTGTTGCCCCTGCAATTGGTGC	
SOE-HP2-F	GCAACATGGCTGCTTGGTGCATCAATGAGCTCTGAAATC	pRS-G(ΔHCPro2)
SOE-HP2-R	CAGAGCTCATTGATGCACCAAGCAGCCATGTTGCAAC	
SOE-tumvHP-1F	CATGGCTGCTTGGTGCACACagtgccgcgggagccaacttc	pRS-G(tuHCPro)
SOE-tumvHP-2R	gctcccgcggcactGTGTGCACCAAGCAGCCATGTTG	
SOE-tumvHP-3F	ctaccgcgttggaggaacaGCATCAATGAGCTCTGAAATC	
SOE-tumvHP-4R	CAGAGCTCATTGATGCtgttcctccaacgcggtagtg	
SOE-GFP-HP2-1F	CATGGCTGCTTGGTGCACACATGAGTAAAGGAGAAGAACTTTTC	pRS-GFP-HCPro2
SOE-GFP-HP2-2R	GACATTTCCTCATCTTGGTGTGCTTTGTATAGTTCATCCATGCCA	
SOE-GFP-HP2-F	GTTCTTCTCCTTTACTCATGTGTGCACCAAGCAGCCATGTTGC	
SOE-GFP-HP2-R	CATGGATGAACTATACAAAGCACACCAAGATGAGGAAATGTC	
SOE-P19-1F	GCTGCTTGGTGCACACAtggaacgagctatacaagg	pRS-G(tbP19)
SOE-P19-1R	gtatagctcgttccaTGTGTGCACCAAGCAGCCATG	
SOE-P19-2F	gcgagGCcAGTAAaGAATTcCAAATGGAtGCATCAATGAGCTCTGAAATC	
SOE-P19-2R	GCaTCCATTTGgAATTCtTTACTgGCctcgctttcttctttgaaggc	
SOE-Strep-HP2-F	cgccgaacctcccgatccacctccggaacctccacctttctcgaactgcgggtggctccaagcggaGTGTGCACCAAGCAGCCATG	pRS-2×Strep-HCPro2
SOE-Strep-HP2-R	gtggaggttccggaggtggatcgggaggttcggcgtggagccacccgcagttcgaaaaagcttccGCACACCAAGATGAGGAAATG	
SOE-Strep-HP2(ΔHP1)-F	CAATTGCAGGGGCAACAATGtccgcttggagccacccgcag	pRS(ΔHCPro1)-2×Strep-HCPro2
SOE-Strep-HP2(ΔHP1)-R	ctgcgggtggctccaagcggaCATTGTTGCCCCTGCAATTG	
SOE-GDD-F	ACTTTGTCTGTAATAATAAATTCACTGTGCACCCTAGT	pRS-G(ΔGDD)
SOE-GDD-F	GCACAGTGAATTTATTATTACAGACAAAGTTAAACATTCC	
8900F*	ATTCCAACAGGGCCAAATAC	RT-PCR detection of ANRSV (targeting *CP*)
9300R*	GGACCATGTTCATACTCACTAACAT	
RS-HCPro-HA(F)	ATATACTCGAGATGGCTACACCATCAAAGGC	pCaM-HCPro1-HCPro2-HA, pCaM-HCPro1-HA, pCaM-HCPro2-HA pCaM-ssHCPro2-HA
RS-HCPro-HA(R)	ATATAGGTACCTTAagcgtagtctgggacgtcgtatgggtaAACTTTAAACTGAAACCAACTTTGATC	
RS-HCPro1-HA(R)	ATATAGGTACCTTAagcgtagtctgggacgtcgtatgggtaAAGTAACCATGTTGCAATTTGTGT	
RS-HCPro2-HA(F)	ATATACTCGAGATGTCACACCAAGATGAGGAAATG	
SS-HCPro2-HA(F)	ATATACTCGAGATGTCATTCATGGAAAGTGAAGGG	
SS-HCPro-HA(R)	ATATAGGTACCTTAagcgtagtctgggacgtcgtatgggtaAACTCTAAATTGAAACCAACTCTG	
pCaM-GFP-HCPro2-F	ATATATCTAGAGCACACCAAGATGAGGAAATG	pCaM-GFP-HCPro2
pCaM-GFP-HCPro2-R	ATATAGGATCCTTATCCAACTTTAAATTGAAACCAAC	
pCaM-GFP-CP-F	ATATAGGATCCATGGATATTGGGGAAGAGAGG	pCaM-GFP-CP
pCaM-GFP-CP-R	ATATAGGTACCTTACATGATCATAGCATGGCCAG	
pCaM-CI-mCherry-F	CTACAAATCTATCTCTCTCGAGATGGCTGACTTCCTTGATGTCG	pCaM-CI-mCherry
pCaM-CI-mCherry-R	CCTTGCTCACCATTCTAGAGAACTCAAGACATTTCTTA	
pCaM-CP-mCherry-F	CTACAAATCTATCTCTCTCGAGATGGATATTGGGGAAGAGAGG	pCaM-CP-mCherry
pCaM-CP-mCherry-R	CCTTGCTCACCATTCTAGACATGATCATAGCATGGCCAG	
pCaM-NbRbCS-mCherry-F	ATATACTCGAGATGGCTTCCTCAGTTCTTTCCT	pCaM-NbRbCS-mCherry
pCaM-NbRbCS-mCherry-R	ATATATCTAGAGTAGCCTTCTGGCTTGTAGGC	
RS-9200F*	GAGCAATGCTTACGGATGGC	real-time RT-qPCR
RS-9350R*	GATCATAGCATGGCCAGTGC	
Actin-145F*	AAAGACCAGCTCATCCGTGGAGAA	
Actin-145R*	TGTGGTTTCATGAATGCCAGCAGC	
TuMV-qPCR-F	ACGCTTGATGCAGGTTTGACAG	
TuMV-qPCR-R	TTGAGTGCCAACTGCTTTTGCC	

PVMV-CP-F	TACACGGGCATTGCAGTCAC	
PVMV-CP-R	TGCTCTTCGCCATCAACCAT	
TelMV-qPCR-F#	AGGTCACCTCTAAGACGTCAGAC	
TelMV-qPCR-R#	GCAGTGTGCCTCTCAGTATCTTC	
NbRbCS-qPCR-F	CAGTTCTTTCCTCAGCAGCAGTTG	
NbRbCS-qPCR-F	GCTGGCAATGGAAGTGATGTCAAG	
NbFNR-qPCR-F	CAACGGTTTGGCATGGCTTTTC	
NbFNR-qPCR-R	TTGTTTGCTCTCTGCTCACTGC	
TRV-GUS-F	ATATAGGATCCTCTGGTATCAGCGCGAAGTCT	pTRV2-GUS
TRV-GUS-R	ATATACTCGAGTAGTTAAAGAAATCATGGAAG	
TRV-NbRbCS-F	ATATAGGATCCCGGCGGAAGAGTGCAATGC	pTRV2-NbRbCS
TRV-NbRbCS-R	ATATACTCGAGTATGCCTTCTTCGCCTCTTC	
TRV-NbFNR-F	ATATAGGATCCATCACGGGTGATGATGCTCC	pTRV2-NbFNR
TRV-NbFNR-R	ATATACTCGAGAATCTTGACCTCTGCTCCAG	
HCPro2-Sfi-F	ATTAACAAGGCCATTACGGCCATGGCACACCAAGATGAGG	pBT3-STE-HCPro2
HCPro2-Sfi-R	AACTGATTGGCCGAGGCGGCCCTAACTTTAAATTGAAACCAAC	
attB1-HCPro1-F	GGGGACAAGTTTGTACAAAAAAGCAGGCTTCATGGCTACACCATCGAAGGCAG	Y2H, MY2H, BiFC and Co-IP (Gateway cloning)
attB2-HCPro1-R	GGGGACCACTTTGTACAAGAAAGCTGGGTCACCAAGCAGCCATGTTGCAAC	
attB1-HCPro2-F	GGGGACAAGTTTGTACAAAAAAGCAGGCTTCATGGCACACCAAGATGAGGAAATG	
attB2-HCPro2-R	GGGGACCACTTTGTACAAGAAAGCTGGGTCAACTTTAAATTGAAACCAACTCTG	
attB1-P3-F	GGGGACAAGTTTGTACAAAAAAGCAGGCTTCATGGCATCAATGAGCTCTGAAATC	
attB2-P3-R	GGGGACCACTTTGTACAAGAAAGCTGGGTCTTGGAACTCTTTCTTCTTATTG	
SOE-P3N-PIPO-F	GCCTCCATGAAAAAAAACATCAAGCAATTTTTAGAC	
SOE-P3N-PIPO-R	AATTGCTTGATGTTTTTTTTCATGGAGGCTCCTTTCCAG	
attB2-P3N-PIPO-R	GGGGACCACTTTGTACAAGAAAGCTGGGTCTCTGCAAGCTTCTTTAAACTG	
attB1-6K1-F	GGGGACAAGTTTGTACAAAAAAGCAGGCTTCATGGCAAAGAGAGAGGAGCCACCA	
attB2-6K1-R	GGGGACCACTTTGTACAAGAAAGCTGGGTCTTGAAACTCTTTACCTCCACC	
attB1-CI-F	GGGGACAAGTTTGTACAAAAAAGCAGGCTTCATGGCTGACTTCCTTGATGTCGAAC	
attB2-CI-R	GGGGACCACTTTGTACAAGAAAGCTGGGTCTTGGAACTCAAGACATTTCTTAAT	
attB1-6K2-F	GGGGACAAGTTTGTACAAAAAAGCAGGCTTCATGTCAGAACAAGTTTTAACAAAAG	
attB2-6K2-R	GGGGACCACTTTGTACAAGAAAGCTGGGTCCTCAAATTCTTTCACAAAATC	
attB1-VPg-F	GGGGACAAGTTTGTACAAAAAAGCAGGCTTCATGGCAGCAAAGAAAAAGAGAGC	
attB2-VPg-R	GGGGACCACTTTGTACAAGAAAGCTGGGTCTTCCATAATTTTTGTCGGCC	
attB1-NIa-Pro-F	GGGGACAAGTTTGTACAAAAAAGCAGGCTTCATGTGCTCAACTGAAGTGCCACAT	
attB2-NIa-Pro-R	GGGGACCACTTTGTACAAGAAAGCTGGGTCCTGAAAGTTTTCAAAATCTTC	
attB1-NIb-F	GGGGACAAGTTTGTACAAAAAAGCAGGCTTCATGATTAGGCGAGTTACACACGTAC	
attB2-NIb-R	GGGGACCACTTTGTACAAGAAAGCTGGGTCTTGAAATTCCTTACTTGCGTG	
attB1-CP-F	GGGGACAAGTTTGTACAAAAAAGCAGGCTTCATGGATATTGGGGAAGAGAG	
attB2-CP-R	GGGGACCACTTTGTACAAGAAAGCTGGGTCCATGATCATAGCATGGCCAG	
attB1-NbRbCS-F	GGGGACAAGTTTGTACAAAAAAGCAGGCTTCATGGCTTCCTCAGTTCTTTCCT	
attB2-NbRbCS-R	GGGGACCACTTTGTACAAGAAAGCTGGGTCGTAGCCTTCTGGCTTGTAGGC	
attB1-NbRbCS(ΔCTP)-F	GGGGACAAGTTTGTACAAAAAAGCAGGCTTCATGATGCAGGTGTGGCCACCAAT	
attB1-NbFNR-F	GGGGACAAGTTTGTACAAAAAAGCAGGCTTCATGGCTACTGCAGTAAGTGCTG	
attB2-NbFNR-R	GGGGACCACTTTGTACAAGAAAGCTGGGTCGTAGACTTCAACATTCCATTG	

Note: The primers indicated with asterisks and wells were designed in previous publications [57,58], respectively.

Reference

1. Wang Y, Shen W, Dai Z, Gou B, Liu H, Hu W et al. Biological and molecular characterization of two closely related arepaviruses and their antagonistic interaction in Nicotiana benthamiana. Front Microbiol. 2021; 12: 755156.
2. Gou B, Dai Z, Qin L, Wang Y, Liu H, Wang L, *et al*. A zinc finger motif in the P1 N terminus, highly conserved in a subset of potyviruses, is associated with the host range and fitness of telosma mosaic virus. J Virol. 2023; 97(2): e01444-22.
