# Supplementary figures and images for: Rubisco small subunit (RbCS) is co-opted by potyvirids as the scaffold protein in assembling a complex for viral intercellular movement
Source: PLoS Pathog. 2024 Mar 4;20(3):e1012064. doi: 10.1371/journal.ppat.1012064 (PMC10939294; doi:10.1371/journal.ppat.1012064)

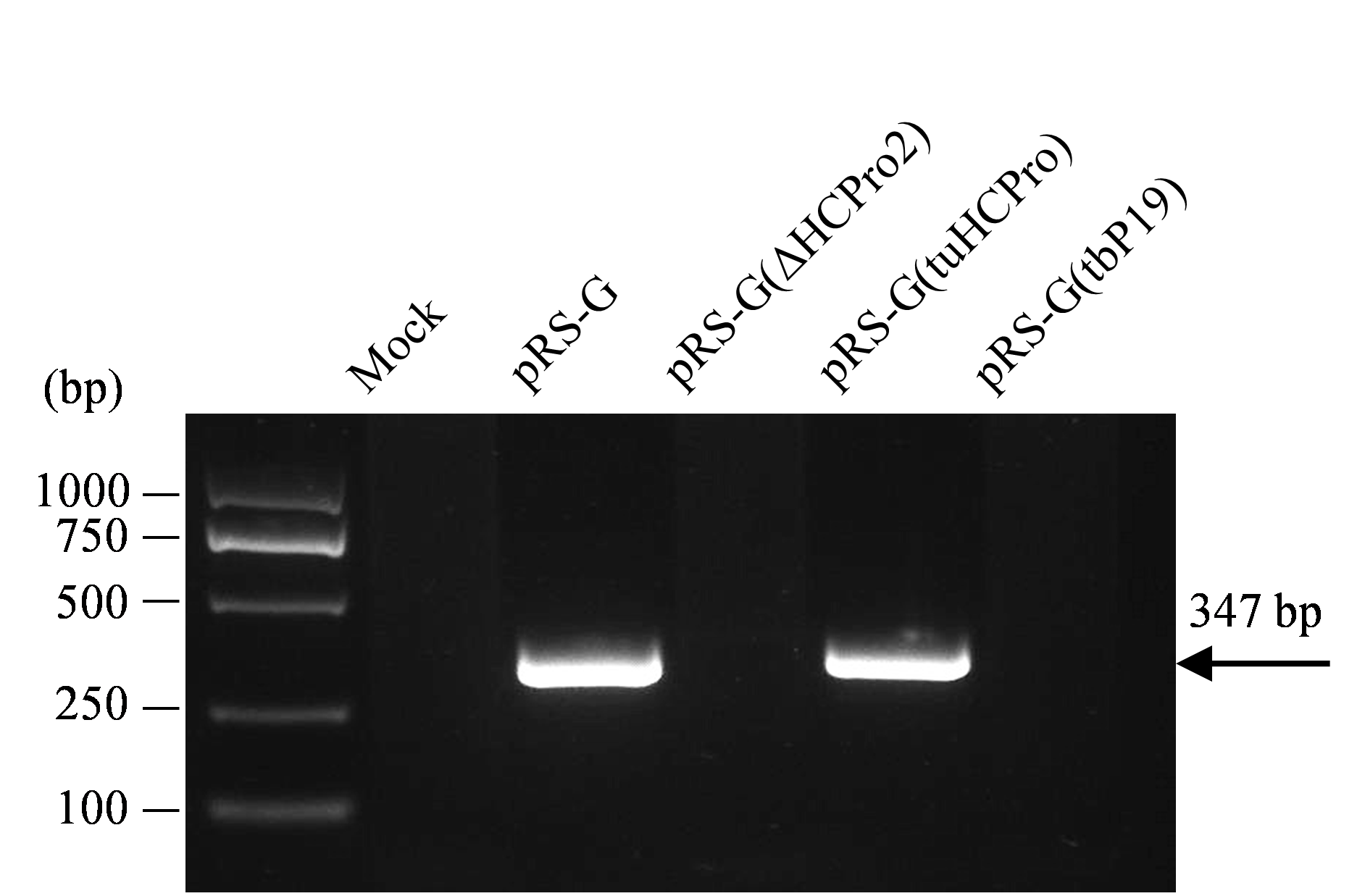

Supplement: S1 Fig — The upper non-inoculated leaves of N. benthamiana plants were assayed at 16 dpi. RT-PCR was conducted with primer set 8900F/9300R (S2 Table) that target viral CP region. (TIF) [file ppat.1012064.s004.tif]

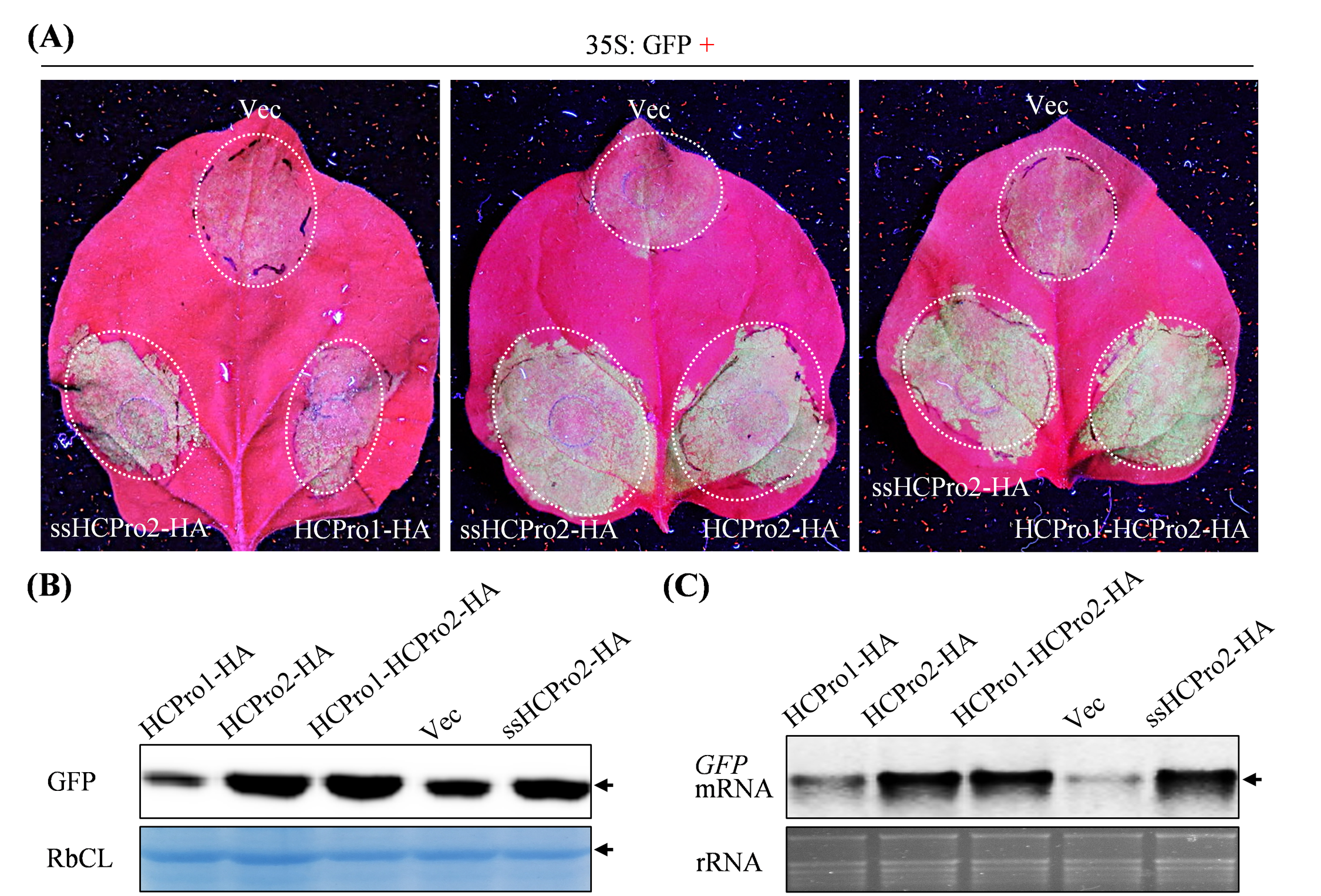

Supplement: S2 Fig — (A) Representative photographs of co-infiltrated N. benthamiana leaves were taken under UV light at 72 hpi. Each of three plasmids (for the transient expression of HCPro1-HA, HCPro2-HA, HCPro1-HCPro2-HA, respectively), together with a GFP-expressing plasmid, were co-inoculated into N. benthamiana leaves via agroinfiltration. Co-expression of GFP along with either empty vector—pCaMterX (Vec) or HA-tagged ANSSV-encoded HCPro2 (ssHCPro2-HA) were included as negative and positive controls, respectively. (B) Immunoblot detection of GFP accumulation in co-inoculated leaf patches at 72 hpi. Coomassie blue staining of RbCL was used as a loading control. (C) Northern blot analysis of GFP transcript accumulation in co-inoculated leaf patches at 72 hpi. Ethidium bromide staining of ribosomal RNA (rRNA) was served as a loading control. (TIF) [file ppat.1012064.s005.tif]

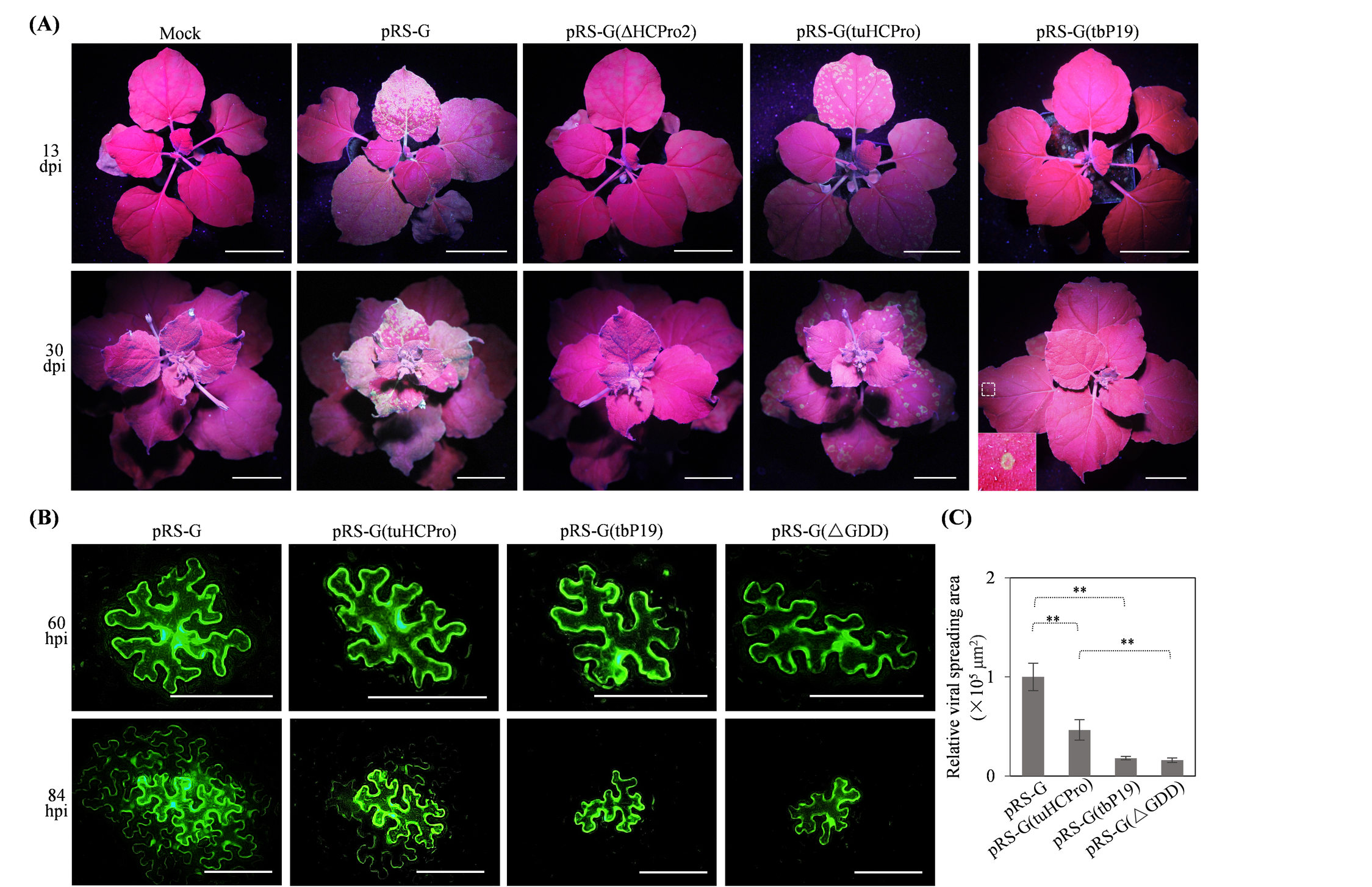

Supplement: S3 Fig — (A) Infectivity test of pRS-G and its derivatives in N. benthamiana. Representative photographs were taken under UV light at 13 dpi and 30 dpi. The leaf region indicated by dashed box is enlarged. Mock, empty vector control. Bars, 5 cm. (B) The observation of viral cell-to-cell movement for the indicated virus clones at 60 hpi and 84 hpi. Bars, 100 μm. (C) Statistical analysis of the size of viral spreading area at 84 hpi. For each clone, at least 25 infection foci from a total of six plants in three independent experiments was analyzed. The size of infection foci is calculated by ImageJ. The data are presented as the mean ± SD (n ≥ 25). The average value for wild-type pRS-G was designated 1×105 μm to normalize the data. **, 0.001<P<0.01. (TIF) [file ppat.1012064.s006.tif]

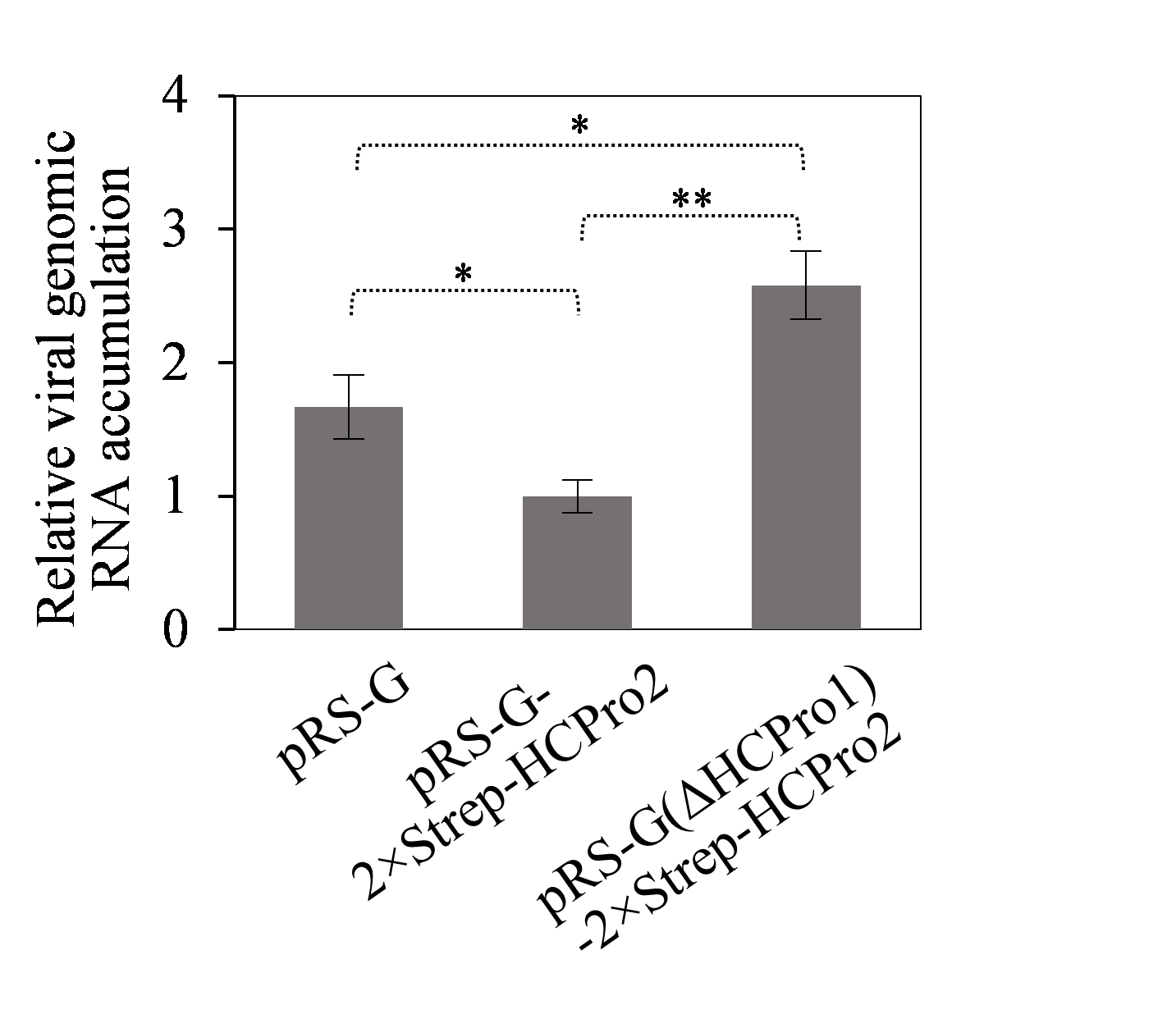

Supplement: S4 Fig — The upper non-inoculated leaves of N. benthamiana plants were sampled at 12 dpi for the assay. RT-qPCR with primer set RS9200F/RS9350R (S2 Table) targeting viral CP region was performed. Error bars denote the SD from three biological replicates. The average value for pRS-G-2×Strep-HCPro2 was designated 1.0 to normalize the data. *, 0.01<P<0.05; **, 0.001<P<0.01. (TIF) [file ppat.1012064.s007.tif]

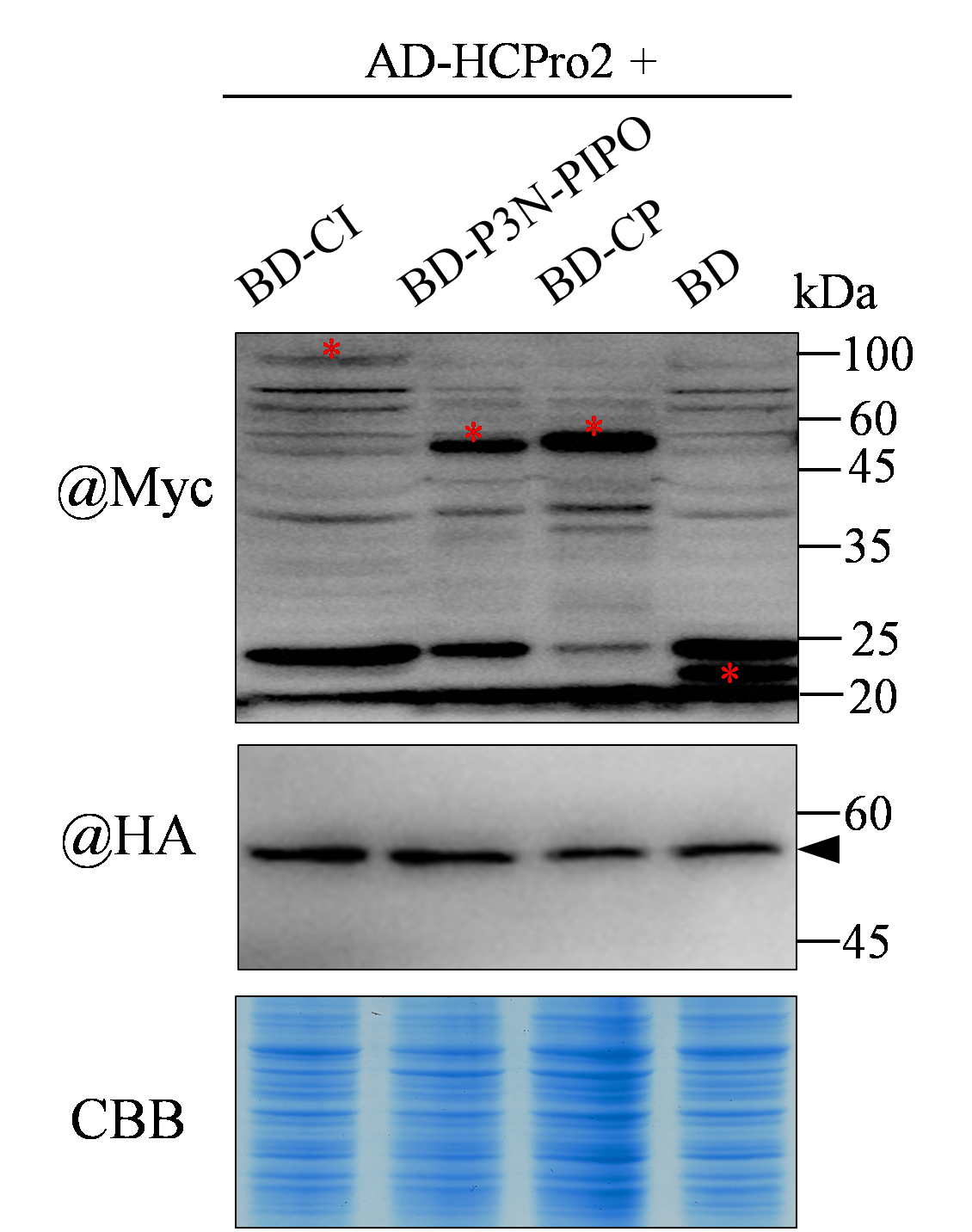

Supplement: S5 Fig — The bands, indicated by red asterisks, correspond to the predicted size of recombinant proteins (~54.5 kDa for AD-HCPro2, 97.87 for BD-CI, 57.46 for BD-P3N-PIPO, and 54.51 for BD-CP). The arrowhead, AD-HCPro2. Coomassie blue staining of the total proteins (CBB) was used as a loading control. (TIF) [file ppat.1012064.s008.tif]

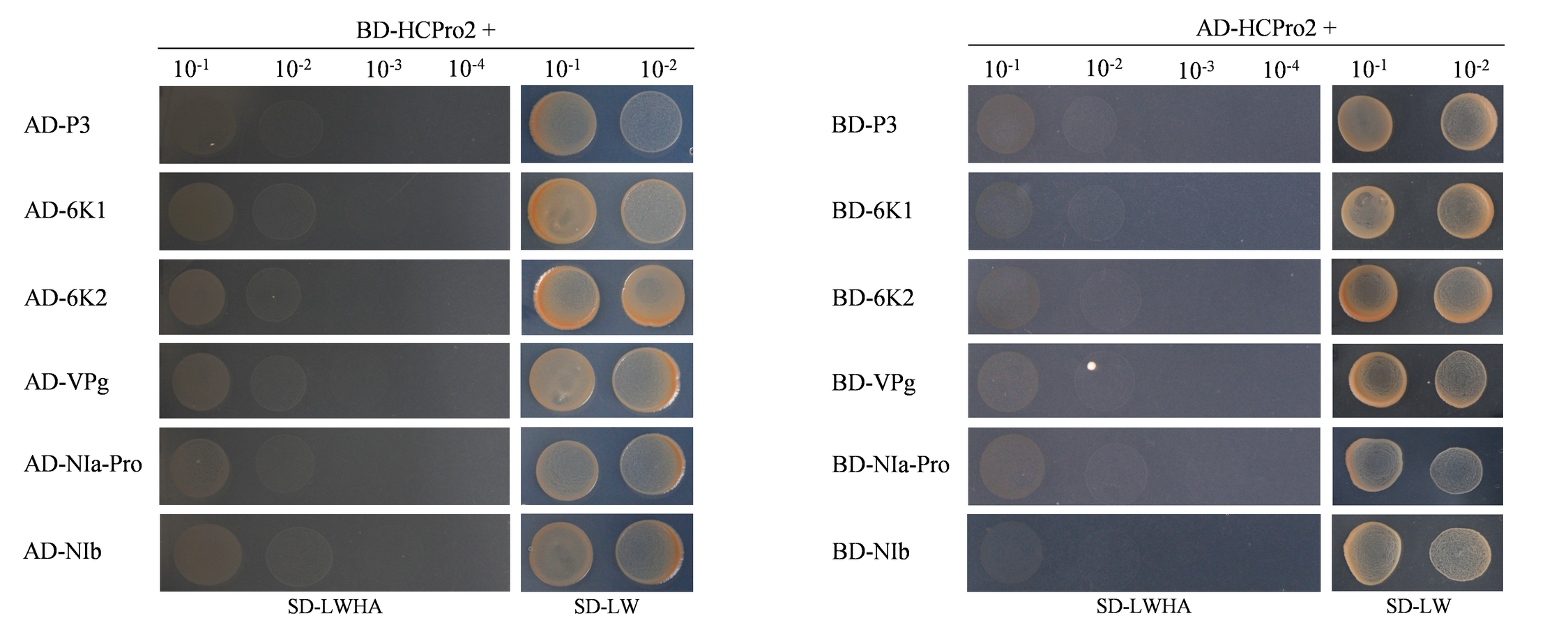

Supplement: S6 Fig — Yeast competent cells (Y2H Gold) were co-transformed to express the indicated pairs of proteins. The transformed cells were subjected to 10-fold serial dilutions and plated on SD/-Trp/-Leu and SD/-Trp/-Leu/-His/-Ade mediums. The plates were cultured at 28°C for four to six days before photographing. (TIF) [file ppat.1012064.s009.tif]

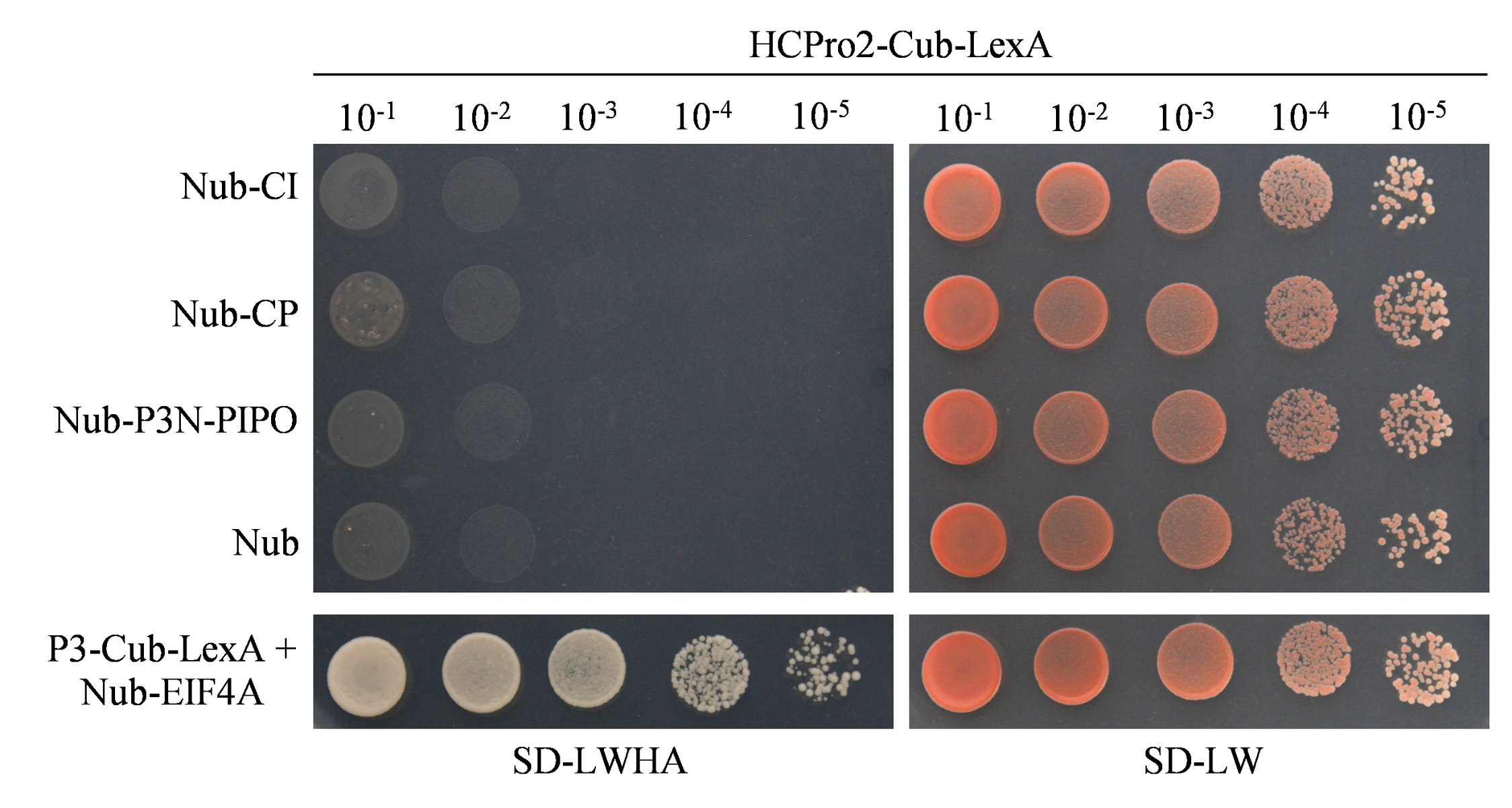

Supplement: S7 Fig — Yeast competent cells (NMY51) were co-transformed to express the indicated pairs of proteins. The transformed cells were subjected to 10-fold serial dilutions and plated on SD/-Trp/-Leu and SD/-Trp/-Leu/-His/-Ade mediums. The plates were cultured at 28°C for four to six days before photographing. Co-transformation of a pair of constructs for simultaneous expression of soybean mosaic virus (SMV) P3-Cub-LexA and Nub-EIF4A [101] was included as the positive control. (TIF) [file ppat.1012064.s010.tif]

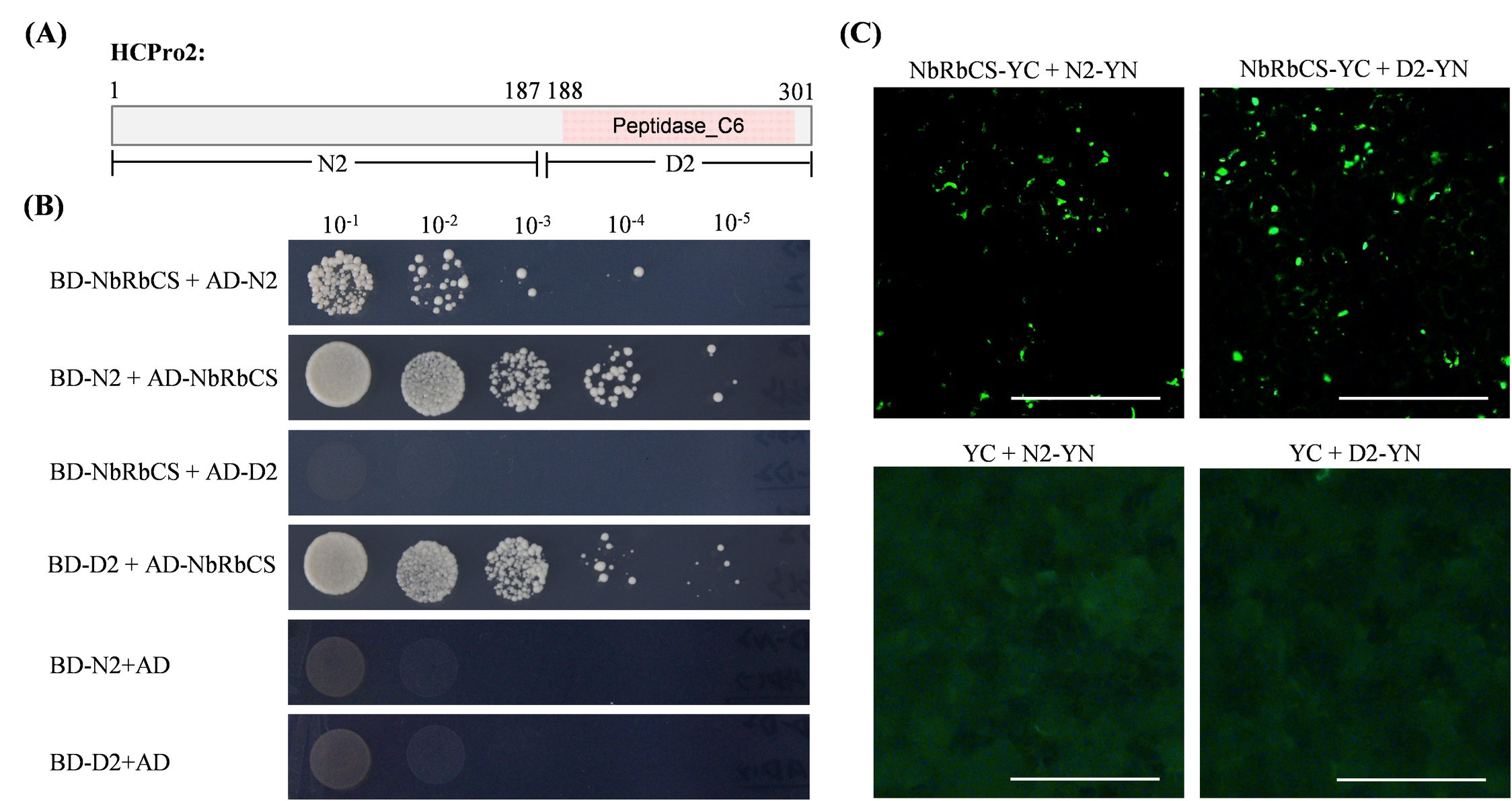

Supplement: S8 Fig — (A) Schematic diagram of HCPro2 showing N2 and D2 domains. The red box represents the cysteine protease domain of HCPro2. (B) The interactions of NbRbCS with N2 and D2 domains were tested by Y2H assays. The coding sequence of NbRbCS was cloned into pGADT7-DEST and pGBKT7-DEST for respective expression of GAL4 AD-fused (AD-NbRbCS) and BD-fused NbRbCS (BD-NbRbCS). The coding sequences of N2 and D2 domains of HCPro2 were cloned into pGADT7-DEST for respective expression of AD-fused N2 (AD-N2) and D2 (AD-D2), and cloned into pGBKT7-DEST for respective expression of BD-fused N2 (BD-N2) and D2 (BD-D2). The co-transformed yeast cells for co-expressing the indicated pairs of proteins / domains were subjected to 10-fold serial dilutions and plated on SD/-Trp/-Leu/-His/-Ade mediums. (C) The interactions of NbRbCS with N2 and D2 domains were tested by BiFC assays. The coding sequences of N2 and D2 were individually integrated into pEarleyGate201-YN for expressing YFP YN-fused N2 (N2-YN) and D2 (D2-YN). N. benthamiana leaves were co-inoculated for the expression of indicated pairs of proteins. YFP signals (shown in green) were observed by fluorescence microscope at 72 hpi. The co-expression of YC and the indicated protein was included as the negative controls. Bars, 100 μm. (TIF) [file ppat.1012064.s011.tif]

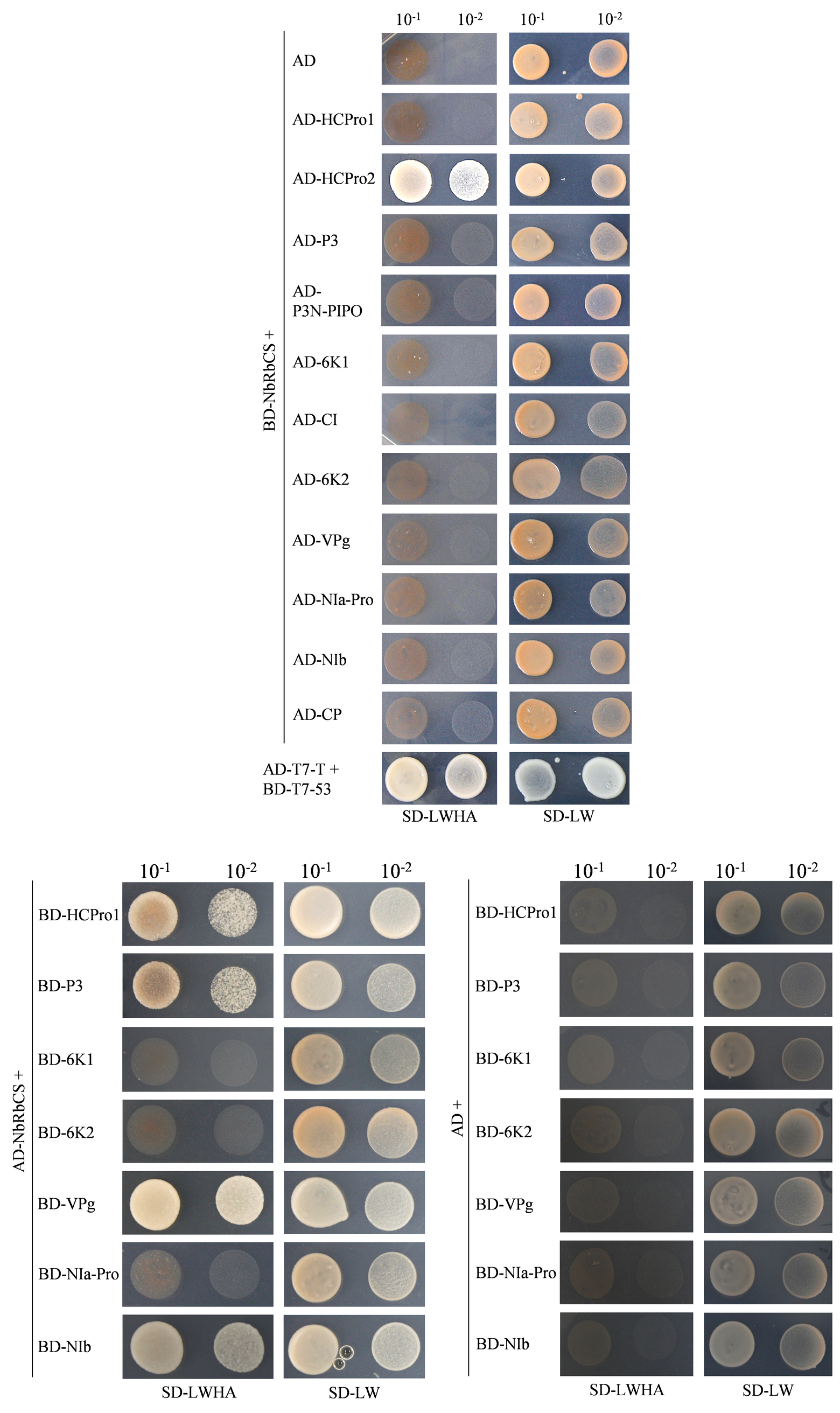

Supplement: S9 Fig — The co-transformed yeast cells for co-expressing the indicated pairs of proteins were subjected to 10-fold serial dilutions and plated on SD/-Trp/-Leu and SD/-Trp/-Leu/-His/-Ade mediums. Co-transformation of yeast cells for simultaneous expression of AD-T7-T and BD-T7-53 was included as the positive control. (TIF) [file ppat.1012064.s012.tif]

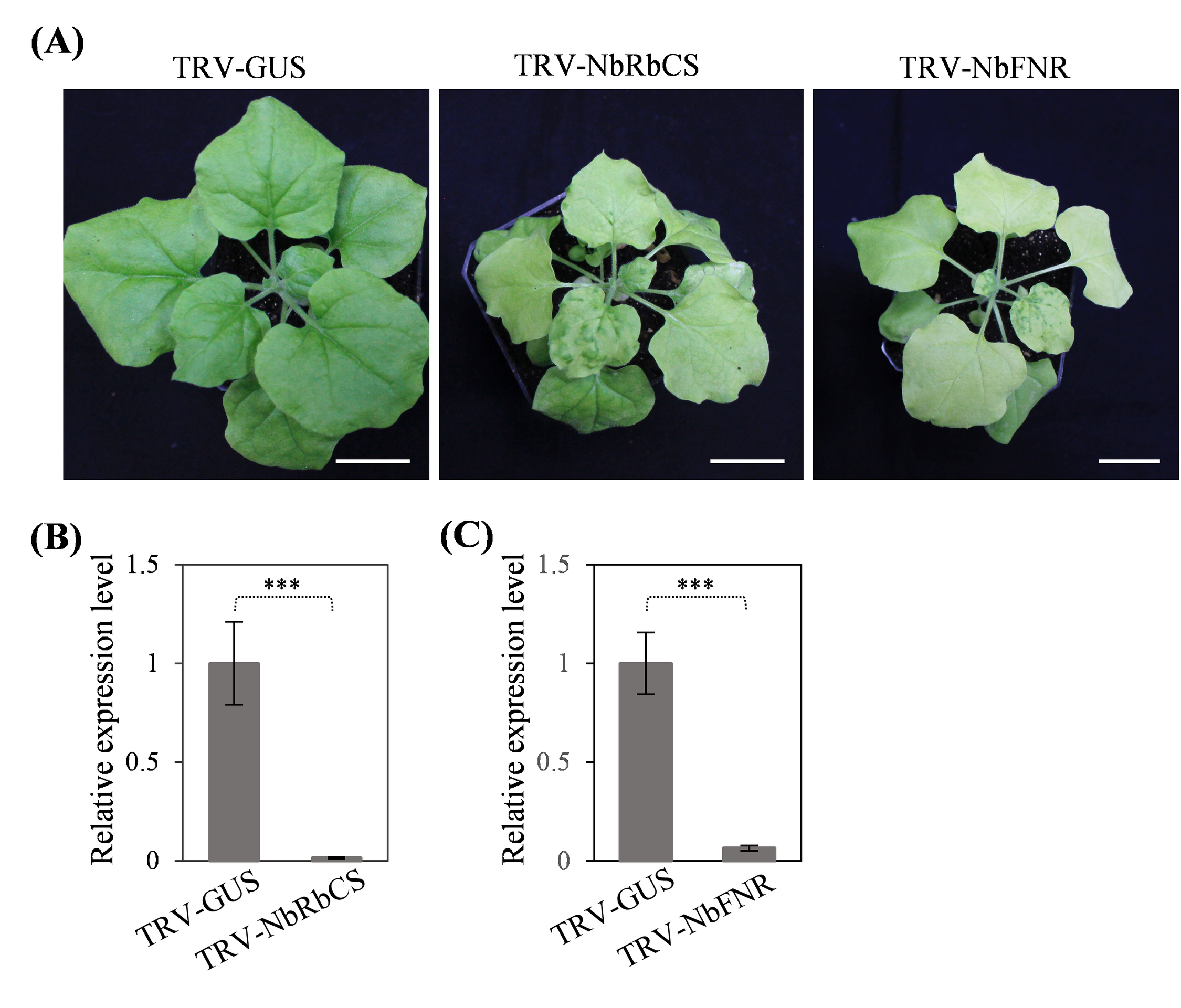

Supplement: S10 Fig — (A) Phenotypic observation of NbRbCS- or NbFNR-silenced in N. benthamiana. N. benthamiana seedlings at 3- to 5-leaf stage were inoculated with pTRV1 along with pTRV2-NbRbCS (TRV-NbRbCS) or pTRV2-NbFNR (TRV-NbFNR), and photographed at 12 dpi. Co-inoculation of pTRV1 and pTRV2-GUS was included as the parallel control. Bars, 2.5 cm. (B, C) Real-time RT-qPCR analysis of NbRbCS or NbFNR mRNA transcript accumulation. The samples were collected at 12 dpi for the assay. Error bars denote the standard errors from three biological replicates. The average value for TRV-GUS was designated 1.0 to normalize the data. ***, P<0.001. (TIF) [file ppat.1012064.s013.tif]

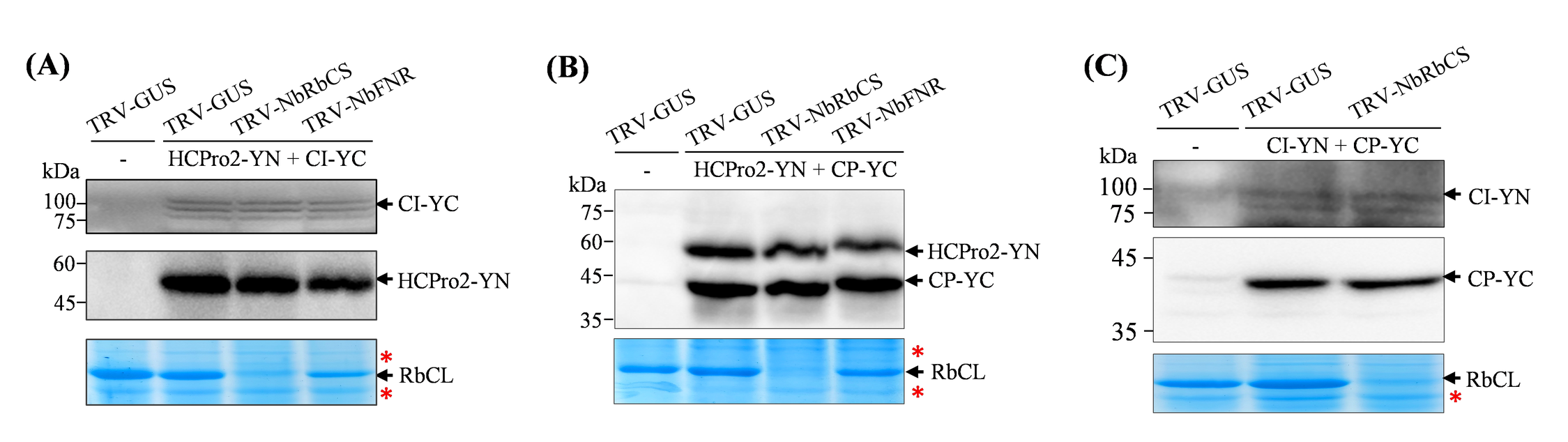

Supplement: S11 Fig — The co-inoculated leaves for co-expression of HCPro2-YN / CI-YC (A), HCPro2-YN / CP-YC (B) or CI-YN / CP-YC (C) were sampled at 60 hpi (B) or 72 hpi (A, C) for immunoblot analysis using anti-GFP antibody. As the abundance of RbCL was greatly decreased along with RbCS-silencing, Coomassie blue staining of protein bands (indicated by red asterisks) was used as a loading control. (TIF) [file ppat.1012064.s014.tif]

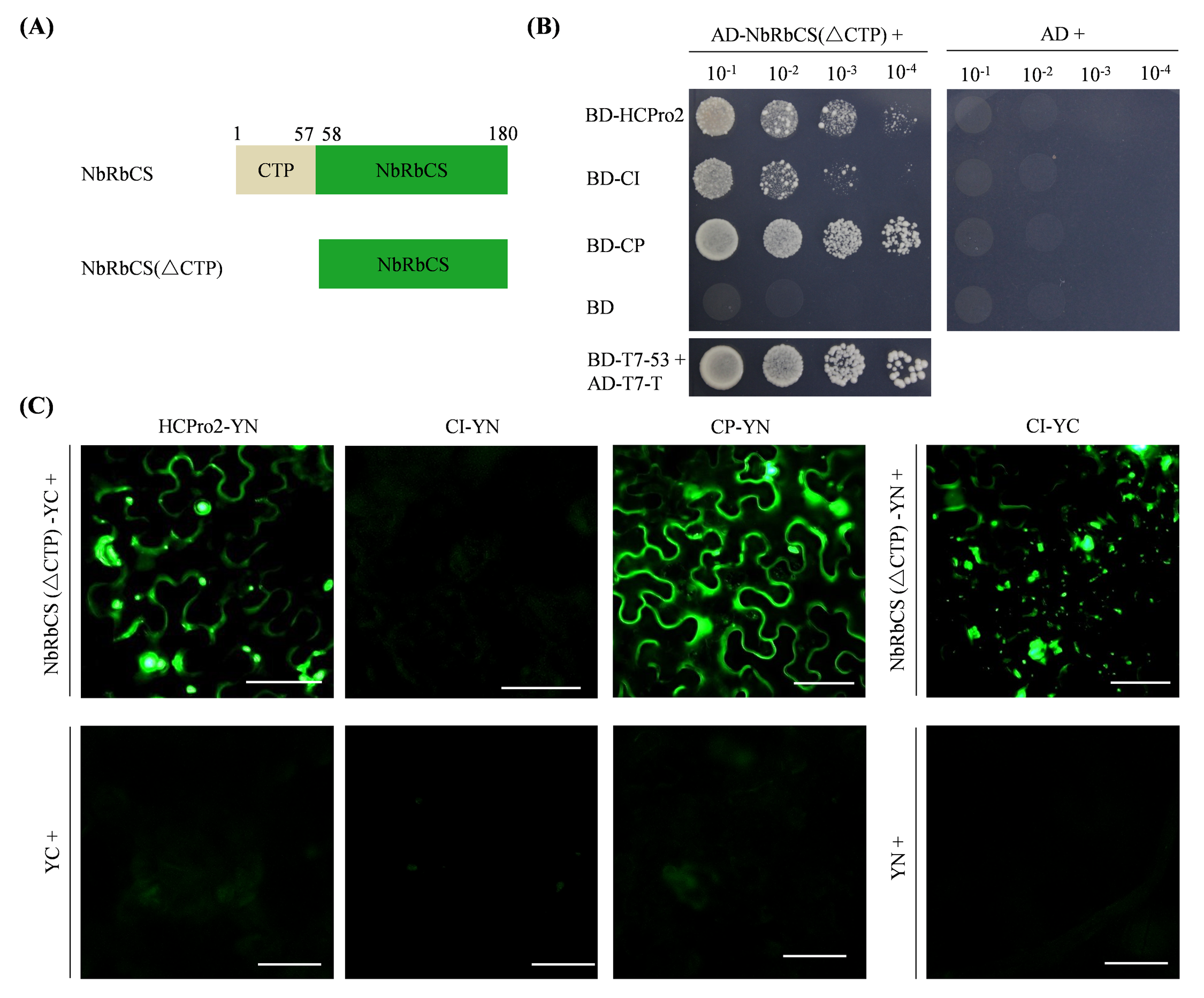

Supplement: S12 Fig — (A) Schematic diagram of NbRbCS(ΔCTP). NbRbCS(ΔCTP) is a truncated version of NbRbCS, with a removal of chloroplast transit peptide (CTP). (B) Y2H tests the interactions of NbRbCS(ΔCTP) with HCPro2, CI and CP. The transformed yeast cells for co-expression of the indicated proteins were subjected to 10-fold serial dilutions and plated on SD/-Trp/-Leu/-His/-Ade mediums. Co-transformation of a pair of constructs for the expression of AD-T7-T and BD-T7-53 was included as the positive control. (B) BiFC assay tests the interactions of NbRbCS(ΔCTP) with HCPro2, CI and CP. N. benthamiana leaves were co-inoculated for the expression of the indicated combination of proteins. YFP signals (shown in green) were observed by fluorescence microscope at 72 hpi. Bars, 50 μm. (TIF) [file ppat.1012064.s015.tif]
